# Nanofeatured Titanium Surfaces for Dental Implants: A Systematic Evaluation of Osseointegration

**DOI:** 10.3390/antibiotics14121191

**Published:** 2025-11-22

**Authors:** Cristina Maria Șerbănescu, Viorel Ștefan Perieanu, Mădălina Adriana Malița, Mihai David, Mihai Burlibașa, Andrei Vorovenci, Camelia Ionescu, Radu Cătălin Costea, Oana Eftene, Ruxandra Stănescu, Mircea Popescu, Florentina Căminișteanu, Liliana Burlibașa

**Affiliations:** 1 Doctoral School, Carol Davila University of Medicine and Pharmacy, 050474 Bucharest, Romania; cristina-maria.serbanescu@drd.umfcd.ro (C.M.Ș.); andrei.vorovenci@rez.umfcd.ro (A.V.); florentina.caministeanu@drd.umfcd.ro (F.C.); 2 Department of Dental Technology, Faculty of Midwifery and Nursing, Carol Davila University of Medicine and Pharmacy, 050474 Bucharest, Romania; viorel.perieanu@umfcd.ro (V.Ș.P.); mihai.david@umfcd.ro (M.D.); radu-catalin.costea@umfcd.ro (R.C.C.); 3 Prosthodontics Residency Program, Department of Prosthodontics, Faculty of Dentistry, Carol Davila University of Medicine and Pharmacy, 050474 Bucharest, Romania; 4 Department of Dental Prosthesis Technology, Faculty of Dentistry, Carol Davila University of Medicine and Pharmacy, 050474 Bucharest, Romania; camelia.ionescu@umfcd.ro; 5 Orthodontics and Dento-Facial Orthopedics Department, Faculty of Dentistry, Carol Davila University of Medicine and Pharmacy, 050474 Bucharest, Romania; oana.eftene@umfcd.ro; 6 Department of Implant-Prosthetic Therapy, Faculty of Dentistry, Carol Davila University of Medicine and Pharmacy, 050474 Bucharest, Romania; 7 Department of Genetics, Faculty of Biology, University of Bucharest, 060101 Bucharest, Romania; liliana.burlibasa@bio.unibuc.ro

**Keywords:** dental implants, nanostructured titanium coatings, osseointegration, bone-to-implant contact (BIC), titanium dioxide nanotubes (TiO_2_ nanotubes)

## Abstract

Background: Whether nanoengineered titanium surfaces confer superior implant stability beyond modern microrough controls remains uncertain. Methods: This systematic review followed PRISMA 2020 guidance: comprehensive multi-database searching with de-duplication; dual independent screening, full-text assessment, and standardized data extraction for predefined outcomes (implant stability quotient [ISQ], mechanical anchorage by removal/push-out/pull-out torque, and histologic bone-to-implant contact). Risk of bias was appraised with RoB 2 for randomized trials, ROBINS-I for non-randomized clinical studies, and CAMARADES (animal experimentation). The certainty of clinical evidence was summarized using GRADE. Results: Across animal models, nanoengineered surfaces consistently improved early osseointegration indices (higher removal torque and bone-to-implant contact at initial healing). In clinical comparative studies, nanoengineered implants showed modest, time-limited gains in early stability (ISQ) versus microrough titanium. By 3–6 months, between-group differences typically diminished, and no consistent advantages were demonstrated for survival or marginal bone outcomes at later follow-up. Methodologic heterogeneity (surface chemistries, timepoints, outcome definitions) and small clinical samples limited quantitative synthesis. Overall, risk-of-bias concerns ranged from some concerns to high in non-randomized studies; the certainty of clinical evidence was low. Conclusions: Nanofeatured titanium surfaces improve early osseointegration but do not demonstrate a consistent long-term advantage over modern microrough implants. Current evidence supports an early osseointegration benefit without clear long-term clinical advantage over contemporary microrough implants. Adequately powered, head-to-head trials with standardized stability endpoints and ≥12-month follow-up are needed to determine whether early gains translate into patient-important outcomes.

## 1. Introduction

Dental implant therapy is a predictable option for replacing missing teeth when contemporary microrough titanium surfaces are used, but the timing and quality of early bone healing remain central to clinical success, especially in immediate placement, immediate or early loading, and low-density bone [1,2]. Over the last two decades, research has progressed from optimizing micron scale roughness to engineering nanoscale features and chemistries that aim to influence the first molecular events at the implant surface [3,4,5]. Reviews and critical appraisals consistently describe improved early osteoblast response, faster bone apposition, and in some cases antibacterial activity with nanofeatured surfaces, while also noting that long-term superiority over current microrough controls is uncertain and inconsistently demonstrated across studies [6,7,8,9,10]. This pattern motivates a focused comparative synthesis that can inform indications where earlier stability may be clinically valuable and where antibacterial effects could lower biologic risk without compromising host response [11].

Several nanostrategies are prominent in the literature and provide a scientific rationale for a comparative evaluation [12]. Titanium dioxide nanotubes created by anodization present ordered pores that concentrate proteins, support osteoblast adhesion, and can serve as reservoirs for ions or drugs; multiple reviews report robust preclinical gains in bone-to-implant contact and mechanical anchorage with more variable clinical data and a need for standardized tube dimensions and surface chemistry [13,14,15]. Nanostructured titanium modulates early protein corona (fibronectin/vitronectin), promotes integrin-mediated adhesion (α5β1/αvβ3), and activates FAK/Src/ERK signaling that supports osteoblast spreading, proliferation, and maturation. Ultra-thin calcium phosphate or hydroxyapatite deposits aim to deliver osteoconductivity while avoiding the delamination seen with early thick plasma-sprayed coatings; systematic summaries suggest the facilitation of early fixation without clear late advantages when compared with optimized microrough titanium [9,16]. Selected nanotopographies and Ca/P-containing chemistries are associated with increased ALP, RUNX2, and OCN expression, consistent with improved early bone-to-implant contact. In parallel, contact-killing nanospikes/graphene and ion-releasing coatings (e.g., Ag, Zn/Mg) can limit early biofilm accrual, provided cytocompatibility is maintained [17]. Carbon-based coatings such as graphene and reduced graphene oxide have been explored for their ability to modulate protein adsorption and cellular signaling while providing intrinsic antibiofilm effects; recent evaluations show promise but also emphasize dose-dependent cytotoxicity risks and the importance of stable integration with the titanium substrate. Additional approaches include trace element incorporation such as strontium, zinc, silver, or magnesium to promote osteogenesis and reduce bacterial viability, ultrafine-grained bulk titanium produced by severe plastic deformation to couple mechanical resilience with hierarchical topography, and catalytic nanozymes that could generate local reactive species to deter biofilm without antibiotics [18].

The microbiologic challenge provides a second pillar for the rationale. Peri-implant diseases are common, and biofilm-driven and antibacterial strategies that act at the initial colonization stage are appealing if they maintain cytocompatibility [19]. Surveys of nano-antibacterial surfaces in dentistry recommend that antimicrobial claims should always be considered alongside host cell data and coating integrity, because surface chemistries that suppress bacteria at the cost of cell viability or that shed particles are unlikely to deliver clinical benefit [12,18,20,21,22,23]. Investigations of silver-depositing or silver-releasing surfaces show consistent antibacterial action but variable effects on osteogenic markers and potential concerns about dose and release kinetics in vivo [24,25,26]. Studies on polymer or non-titanium substrates map design spaces and boundary conditions that are relevant when translating antibacterial concepts to titanium dental implants.

Safety and standardization are recurring themes across reviews and excluded sources, highlighting the need for careful comparative assessment. Overviews of titanium and its corrosion products emphasize vigilance regarding particulate and ionic release under functional load, the potential for inflammatory responses, and the need for long-term surveillance when new layers are introduced at the interface [27]. Broader narratives on nanomaterials in dentistry warn that biological effects are sensitive to particle size, crystallinity, and surface energy, and that in vitro findings can be misleading if evaluated outside of the clinical delivery context [20,28]. Manufacturing-focused summaries of laser texturing and additive methods highlight that process parameters, post-processing, and surface cleanliness materially shape protein adsorption and cell response, and they call for harmonized reporting of surface characterization, including roughness across spatial scales, wettability, chemistry, and residual contaminants.

Despite a sustained expansion of nanoplatforms, the clinical evidence base remains uneven. Human trials are few, sample sizes are modest, follow-up is often short, and outcomes vary from histologic surrogates in preclinical models to stability measures and radiographic parameters in early clinical phases. Several lines of evidence suggest that nanofeatures may shift the kinetics of healing rather than the eventual plateau, which implies that time of assessment is a key moderator and that clinical value may concentrate in situations where early stability dictates treatment plans [6,7,9,29]. At the same time, excluded comparative experiments and translational studies extend the mechanistic picture. For example, laser microtextured titanium surfaces and directionally processed titanium have been reported to affect cell alignment, attachment strength, and early mineralization, but these gains do not uniformly translate to later structural outcomes in vivo and depend heavily on precise surface metrics and cleanliness [11,30,31,32,33]. Drug or growth factor loading into nanotube arrays shows strong osteogenic signals in animals yet raises design questions about controlled release, local concentration, and the balance between promoting bone growth and avoiding soft tissue irritation [29,34,35]. These heterogeneous findings reinforce the need to compare nanocoated titanium with suitable microrough controls using consistent osseointegration endpoints and to interpret any antibacterial gains within the same framework.

Finally, systematic and narrative reviews that evaluate clinical success rates and marginal bone changes across mixed implant surfaces report high overall survival with contemporary titanium and incremental differences between surface types, adding a pragmatic perspective to the existing literature [8,36,37]. In general, the literature supports a clear but nuanced rationale. Nanofeatured surfaces tend to improve early markers of osseointegration and can provide antibacterial activity, yet the size, timing, and durability of these benefits relative to advanced microrough controls remain as open questions for clinicians. The balance between efficacy and safety requires explicit attention to coating integrity, ion or particle release, and standardized reporting of surface properties and biologic endpoints.

The aim of the review is to determine whether nanocoated or nanostructured titanium dental implant surfaces improve osseointegration compared with conventional titanium surfaces in controlled human and preclinical studies, while documenting antimicrobial and cytocompatibility outcomes that influence its translation to clinical practice.

## 2. Materials and Methods

### 2.1. PICO Process Design

The research question is as follows: “In subjects receiving dental implants (including human clinical recipients, in vivo animal models of implant placement, and relevant in vitro/laboratory models), do nanocoated or nanoscale surface-modified titanium implant or abutment surfaces, compared with conventional titanium surfaces (e.g., machined or grit-blasted/acid-etched), improve osseointegration outcomes such as bone-to-implant contact, bone density, removal/reverse torque, and related histomorphometric or stability measures?”. The PICO process is presented in Table 1.

### 2.2. Search Strategy

This systematic review was conducted in accordance with the PRISMA 2020 guidelines [38] (the completed PRISMA checklist is provided in the Supplementary Materials). The selection of studies was conducted using the following electronic databases: PubMed-Medline, Scopus, and Web of Science. Articles published from January 2016 to August 2025 were identified using a combination of controlled vocabulary and free-text terms representing three concepts: (i) dental/oral implants, (ii) nanoscale structures or coatings (e.g., nanoparticles, nanotubes, nanomaterials, nanocoatings), and (iii) osseointegration outcomes, including bone-to-implant contact (BIC). Exact database strings are provided in Table 2. Searches were limited to English-language original research articles; reviews and conference abstracts/proceedings were excluded at the search or screening stage. When preliminary results yielded substantial orthopedic implant records, we increased specificity by adding dentistry-context terms (e.g., dentistry, oral, periodontal) to the query. In addition to database searches, we ran a supplementary search using Elicit (Ought, Oakland, CA, USA) to increase recall. Elicit queries the Semantic Scholar corpus (via the Semantic Scholar API), retrieving titles/abstracts (and full text when available) for the top results, with optional filters for date and study type. We used the same three-concept keyword set (implants + nanoscale structures/coatings + osseointegration/BIC), screened the returned records, and de-duplicated against the bibliographic databases before the eligibility assessment. We considered clinical, in vivo animal, and in vitro/laboratory models within dental implantology. The eligibility assessment prioritized titanium-based substrates, controlled designs, and osseointegration-relevant outcomes; non-comparative reports, studies of non-titanium implants without titanium comparators, and review-type publications were excluded (Table 3). Comparative studies whose test implants were made exclusively of non-titanium materials (e.g., zirconia) were not included, even when a titanium comparator was present, unless a nano-modified titanium arm allowed the extraction of titanium-based data. In mixed-material experiments, only titanium arms were analyzed. Coatings fabricated on titanium using, for example, zirconia particle blasting or zirconia-derived nanophases, remained eligible because the substrate was titanium. All borderline decisions were screened in duplicate and adjudicated before inclusion to preserve reproducibility and alignment with the a priori criteria. Data extraction captured study design/model, nanosurface description, comparator surface, and osseointegration endpoints and timepoints. This review spans clinical, in vivo animal, and in vitro evidence to map the translational arc from mechanism to clinical surrogates. Preclinical studies inform biological plausibility (surface–protein–cell interactions and early bone formation) but were not used to infer patient-level effects. To manage heterogeneity arising from models, coating chemistries, outcomes, and timepoints, we prespecified narrative synthesis and segregated results by model.

### 2.3. Risk of Bias and Reporting Quality Appraisal

The risk of bias was appraised using design-specific tools. For human randomized trials, risk of bias was assessed with Cochrane RoB 2 [39]. For human non-randomized clinical studies, we used ROBINS-I to judge bias relative to a hypothetical target trial. In vitro studies were appraised with RoBDEMAT [40]. Because not all animal experiments in our set were randomized controlled designs, animal studies were appraised primarily with the OHAT risk-of-bias framework (which accommodates randomized and non-randomized designs and yields domain judgments that roll up to a Tier 1–3 study-confidence classification) [41]. We also performed a SYRCLE [42] assessment as a sensitivity analysis.

To complement internal validity, reporting/reliability in animal studies was evaluated using the CAMARADES (The Collaborative Approach to Meta-Analysis and Review of Animal Data from Experimental Studies) 10-item checklist (yes/no items such as randomization, blinding, sample size calculation, temperature control, welfare compliance; reported descriptively and not used for weighting). Given the bench-to-animal nature of nanocoatings, we additionally mapped CRIME-Q domains (Quality of Reporting, Methodological/Technical Quality, and Risk of Bias) to provide context on surgical technique, implant handling, and perioperative regimens; CRIME-Q outputs are presented narratively alongside OHAT and CAMARADES. For preclinical nano–bio work (both in vitro and in vivo), MIRIBEL (Minimum Information Reporting in Bio–Nano Experimental Literature) elements (material characterization, biological characterization, protocol details) were applied to document nano-specific reporting; for animal studies, ARRIVE 2.0 (Animal Research: Reporting of In Vivo Experiments) Essential 10 items were also checked [43]. Reporting checklists (MIRIBEL, ARRIVE, CAMARADES, CRIME-Q) were used to describe reporting quality and technical rigor, not to derive pooled risk-of-bias scores.

Four reviewers independently applied all tools using piloted forms; disagreements were resolved by consensus. Per our prespecified rule, when a safeguard required by a tool was not explicitly reported, we judged that domain as “probably high” (OHAT “−”) or “some concerns/high” according to the instrument, rather than defaulting to “unclear.” Domain tables were generated for transparency.

### 2.4. Assessing Certainty of Evidence

The certainty of evidence was appraised the using GRADE (Grading of Recommendations Assessment, Development, and Evaluation), applied only to human comparative studies (RCTs and non-randomized); animal and in vitro evidence informed biological credibility but was not graded. The patient-important outcomes were specified: marginal bone loss (MBL), soft tissue indices (mPI/mGI/BOP), early infection/peri-implant mucositis, implant survival/success, and microbiological surrogates (treated as indirect). Certainty started high for RCTs and low for non-randomized studies, then was downgraded across five domains: risk of bias (using RoB 2 for RCTs; ROBINS-I for non-randomized), inconsistency (direction/overlap of effects; pooling only when appropriate), indirectness (population/intervention/comparator/outcome and surrogate endpoints), imprecision (95% CIs relative to minimally important differences and event scarcity; no optimal information size/TSA analyses reported), and publication bias (small-study effects and preregistration/registry checks when available).

For animal evidence, the results were not statistically combined with clinical outcomes. Instead, body-of-evidence confidence using OHAT Tiers (1–3) and reported CAMARADES (reporting/quality) and CRIME-Q (method/technical domains) were summarized descriptively. To support translational interpretation, the preclinical outcomes were mapped (e.g., BIC, pull-out strength, early bone volume) to the most proximal clinical surrogates (e.g., early marginal bone change, soft tissue indices), and this linkage was treated as a biological probability rather than direct effect evidence.

Quantitative meta-analysis was not undertaken due to substantial methodological heterogeneity across studies, including (i) mixed designs (human, animal, in vitro) with non-comparable endpoints; (ii) variable outcome definitions and timepoints (e.g., ISQ at differing days/weeks; BIC at disparate healing intervals); (iii) inconsistent or missing variance data (SD/SE/CI) required for pooling; (iv) diverse measurement platforms (removal torque vs. push-out vs. histomorphometry with differing regions of interest); and (v) heterogeneous surface descriptions and incomplete characterization domains. In line with the protocol, evidence was, therefore, synthesized narratively with stratification by evidence tier and outcome domain.

### 2.5. Reviewers and Use of Automation and AI-Assisted Tools

Titles/abstracts and full texts were screened in duplicate by four independent reviewers. Data extraction was likewise performed in duplicate. Any discrepancies at either stage were first discussed between the paired reviewers; unresolved conflicts were adjudicated by two senior reviewers serving as independent arbiters. All inclusion/exclusion decisions, risk-of-bias judgments, and final data entries required human consensus. Elicit Pro (Ought, Oakland, CA, USA) supported citation discovery and relevance triage, and EndNote X9 was used for reference management and de-duplication. ChatGPT 5.0 (OpenAI, San Francisco, CA, USA) was employed only to draft standardized, non-decisional text (e.g., table captions, Methods phrasing), to improve readability, to generate schematic/illustrative figures, and to help structure extraction templates. AI outputs were not used to make eligibility decisions, perform risk-of-bias assessments, populate data fields, derive numerical values, or finalize study characteristics without human verification. A structured validation protocol was applied to all AI-assisted content: (i) line-by-line human editing by two reviewers; (ii) cross-checking of any factual statements, study attributes, and numbers against the extraction sheets and original source PDFs; (iii) rejection of any AI-suggested citations not traceable to the registered search sources; and (iv) retention of an audit trail (prompts and outputs archived with timestamps) to enable reproducibility. Final acceptance of AI-assisted text or graphics required explicit agreement by two human reviewers.

## 3. Results

### 3.1. Selection of Studies

**Figure 1:**
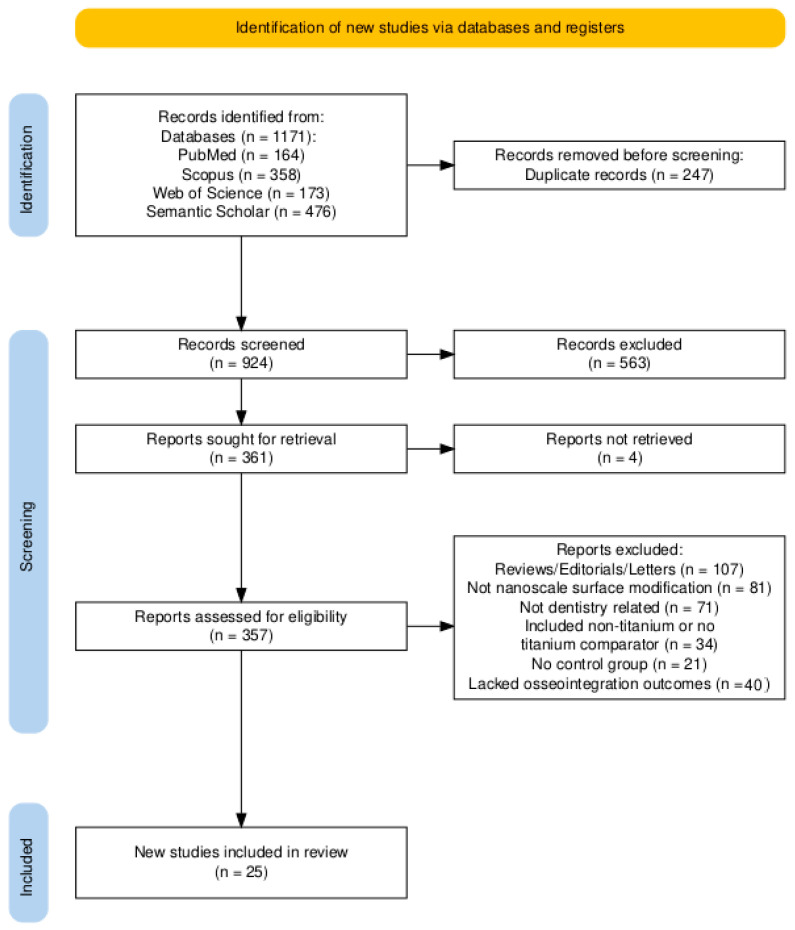
PRISMA flow diagram.

The search yielded 1171 records in total (PubMed, *n* = 164; Scopus, *n* = 358; Web of Science, *n* = 173; Semantic Scholar, *n* = 476). After de-duplication in EndNote X9 (*n* = 247), 924 records were screened at title/abstract. A total of 563 records were excluded at this stage, 361 reports were sought for full-text review, and 4 could not be retrieved, leaving 357 full texts assessed for eligibility in duplicate. A total of 331 reports were excluded due to the following reasons: most commonly review/editorial/letter/protocol/no extractable data (*n* = 107), not nanoscale surface modification (*n* = 81), and not a dental implant context (*n* = 71); other reasons included non-titanium or no titanium comparator (*n* = 34), no comparator/control group (*n* = 21), and lacked osseointegration outcomes (*n* = 40). In total, 25 studies met the inclusion criteria and were included in the qualitative synthesis: human clinical comparative (*n* = 4; RCTs *n* = 2; non-randomized *n* = 2), in vivo animal-controlled studies (*n* = 10), in vitro/laboratory studies (*n* = 1), and combined in vitro/in vivo studies (*n* = 10) (Figure 1). No meta-analysis was planned or conducted; findings were synthesized narratively. Four reviewers screened independently; inter-rater agreement before adjudication was Fleiss’ κ = 0.72 (95% CI 0.68–0.76) at title/abstract and 0.81 (0.77–0.85) at full text. A total of 18% of records required adjudication by two senior reviewers; disagreements were resolved by consensus.

### 3.2. Risk of Bias and Reporting Quality

Human clinical studies: Both randomized trials were judged overall to have “some concerns” using RoB 2, driven mainly by incomplete detail on deviations from intended interventions and handling of missing data; outcome measurement was blinded and judged to have low risk (Table 4). Non-randomized clinical studies ranged from moderate to serious risk with ROBINS-I due to potential confounding and limited reporting of masking and preregistration, while intervention classification and outcome measurement were generally appropriate (Table 5). Animal studies: Across the animal experiments, most domains related to exposure characterization (implant/coating identification and preparation) and outcome assessment (standard histomorphometry/µCT) were rated probably/definitely low. In contrast, randomization, allocation concealment, and blinding of personnel/assessors were often not described and, therefore, judged to be probably high by rule. Overall, OHAT confidence categorized four studies as Tier 2 and 13 studies as Tier 3; none met Tier 1 (Table 6). A SYRCLE sensitivity check mirrored these patterns, with the same critical gaps centered on randomization and blinding rather than data completeness.

In vitro studies (RoBDEMAT): Bench studies commonly provided clear specimen preparation and outcome validity, but randomization/blinding of specimens and a priori sample size calculations were rarely reported, leading to “some concerns” in most domains (Table 7).

**Figure 2:**
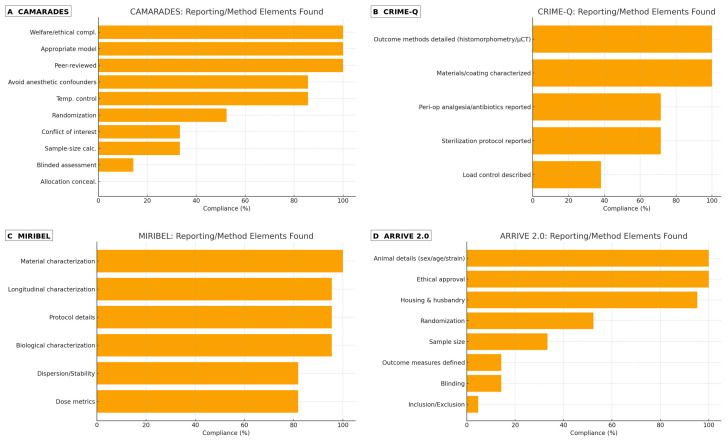
Reporting and methodological elements detected across preclinical studies. Panels: (**A**) CAMARADES (animal reporting/quality), (**B**) CRIME-Q (technical/method elements), (**C**) MIRIBEL (nano–bio reporting, animal + in vitro), and (**D**) ARRIVE 2.0 (animal reporting). Bars display the percentage of studies reporting each item. Items not explicitly reported were counted as absent.

Reporting and technical quality (CAMARADES, CRIME-Q, MIRIBEL, ARRIVE 2.0): The most frequently fulfilled CAMARADES items were peer-reviewed publication, appropriate model, and welfare/ethical compliance; the least frequently fulfilled were sample size calculation, randomization, blinding, and temperature control reporting. CRIME-Q mapping highlighted strong materials/implant descriptions and sterilization protocols in a subset, with heterogeneity in surgical technique standardization, load control, and perioperative analgesia/antibiotics reporting. MIRIBEL mapping showed that surface morphology/roughness/thickness/composition were usually characterized (e.g., SEM/AFM/EDS), whereas dose metrics (delivered vs. administered), dispersion/stability in relevant media, and longitudinal characterization were inconsistently reported. ARRIVE 2.0 checks reiterated shortfalls in randomization, blinding, sample size justification, and prespecified inclusion/exclusion criteria, which likely contributed to the OHAT Tier 3 designations (Figure 2).

Implications for synthesis: Taken together, the clinical body of evidence is limited by sample size and some concerns about trial conduct but benefits from blinded outcome assessment; the animal evidence is broadly Tier 2–3 due to reporting omissions in key internal validity safeguards; and in vitro evidence is constrained by typical laboratory omissions (randomization/blinding/power).

### 3.3. Evidence Power (Clinical vs. Preclinical)

Using GRADE, the evidence power is limited across patient-important outcomes. For marginal bone loss and soft tissue indices, certainty is low: both RCTs carried some concerns for bias, and, critically, the total sample across trials (~75 participants) does not meet the optimal information size for plausible minimally important differences (≈0.2–0.3 mm) under typical SDs (≈0.4–0.6 mm), yielding serious imprecision. For early infection/peri-implant mucositis and implant survival/success, certainty is very low due to very serious imprecision (rare events, short follow-up) and study limitations; with only two trials, inconsistency is not estimable, and heterogeneous definitions/timepoints further limit pooling. Indirectness also affects microbiological surrogates, which were not considered direct patient-important outcomes. No upgrading criteria were met (no large, consistent effects or dose–response, and residual confounding would more likely attenuate than exaggerate benefit). Publication bias is plausible given small studies and limited preregistration. Overall, by GRADE, current clinical evidence is insufficient to claim superiority of nanocoated over uncoated implants; additionally, adequately powered, preregistered RCTs with standardized outcomes are needed (Table 8).

Preclinical evidence is abundant but indirect. Most animal studies were OHAT Tier 2–3, chiefly due to unreported randomization/blinding despite good exposure and outcome characterization. This body of work supports the biological plausibility for nanocoatings (osseointegration proxies and antimicrobial activity), but its indirectness to patient-important outcomes means it cannot resolve the clinical effect on its own. As a result, the overall certainty of clinical evidence remains low to very low, and further adequately powered, well-reported RCTs are needed before clinical adoption decisions can be made.

### 3.4. Summary of Included Studies

Twenty-five studies met the inclusion criteria, encompassing a range of experimental models and nanocoating strategies. Four studies were in vivo human trials, including two randomized controlled trials and two observational or exploratory studies of patients. Ten were in vivo animal studies (e.g., rabbit, rat, dog, sheep, or minipig models), another ten were combined in vitro and in vivo experiments (often testing cellular responses in vitro alongside animal implantation), and one was a stand-alone in vitro study evaluating osteogenic and antibacterial performance under dynamic oral-flow conditions [49] (Table 9). The follow-up periods for bone healing were typically short-to-medium term: most animal studies evaluated early osseointegration at 2–12 weeks post-implantation, while the human studies assessed outcomes from immediate loading up to about 4–6 months.

Across the included studies, the control implants were uncoated titanium surfaces with standard topographies, most commonly conventional sandblasted/acid-etched (SLA) or machined surfaces. Several control groups were well-established commercial surfaces (e.g., machined cp-Ti or SLA without nanocoating). The nanocoated interventions varied widely in composition and fabrication. Approximately one-quarter of the studies (6/25) examined titanium dioxide nanotube surfaces created by anodization, sometimes with added treatments [50,55,57,59,60,61,69]. Hydroxyapatite (HA) nanoparticle coatings were tested in three studies [44,56,64,66]. Silver-based nanocoatings (typically Ag nanoparticles or Ag ion incorporated into TiO_2_) were evaluated in two studies [48,67]. Other nanoscale modifications included strontium-infused surfaces, nanopatterned or nanostructured topographies on titanium, and unique coatings such as nanospikes produced by acid peroxide treatment, ultrananocrystalline diamond, nanofibrous polymer/ceramic layers, bioactive agent-loaded nanostructures like propolis in TiO_2_ nanotubes or Korean ginseng in nanotubes, and dual ion implantation of zinc and magnesium. Despite this diversity, all coatings were applied onto titanium implant substrates and aimed at nanoscale surface features or chemistry to enhance integration.

Almost all studies measured multiple osseointegration outcomes. The most common outcome was histomorphometric bone-to-implant contact (BIC), quantified in 17 of the 25 studies as a percentage of implant surface in direct bone contact. Roughly ten studies assessed bone density or bone volume around implants (via histology or micro-CT), and about seven measured related metrics such as bone area fraction or bone volume/tissue volume. Four studies evaluated removal torque or push-out strength as a biomechanical measure of integration (typically in animal models). A few studies reported bone formation rates or mineral apposition (using fluorescent labels), and three reported bone mineral content/density in the peri-implant region. Several in vitro components measured osteogenic activity markers: six studies quantified alkaline phosphatase (ALP) activity or mineralized nodule formation in cell culture, and five examined osteogenic gene expression (e.g., RUNX2, osteocalcin, BMPs) to gauge cellular differentiation. Only a small subset specifically included antimicrobial or biofilm, and one human trial uniquely focused on soft tissue outcomes (healing and peri-implant mucosal health) with a nano-modified abutment. Importantly, most studies reported on early osseointegration phases; none had clinical follow-up beyond 1 year, and long-term functional outcomes (such as implant survival) were outside the scope of these experiments. To minimize interpretive bias across heterogeneous designs, results are signposted by evidence tier—first, human clinical studies, followed by animal in vivo experiments, and then in vitro assays—and within each tier by outcome.

### 3.5. Osseointegration Evaluation

Across the included evidence, nanoengineered surfaces generally improved early osseointegration metrics (most clearly BIC and, in a subset of clinical series, ISQ), while insertion and removal torque were usually unchanged once integration had consolidated. The direction and magnitude of effects varied by coating class, animal model versus human setting, and follow-up time, with several studies showing pronounced early gains that attenuated as both test and control surfaces matured (Table 10).

#### 3.5.1. Clinical Studies

In humans, early stability favored nano-modified implants in some trials. For example, Ko et al. (2024) reported higher ISQ for hydroxyapatite nanocoated SLA implants during healing, with mean ISQ ~76.5 vs. ~71.3 at 2 months and ~79.1 vs. ~73.4 at 4 months (both significant), despite similar radiographic bone levels, consistent with an interface quality effect rather than bulk bone gain [44]. In a clinical series with a silver nanoparticle surface, Memon et al. (2025) observed significantly greater BIC than uncoated controls (*p* = 0.013), alongside reductions in local inflammatory markers, indicating that antibacterial functionality did not compromise integration [67]. In contrast, an anodized abutment surface study [45] did not detect stability or bone advantages over conventional components, underscoring that not every nanofeature translates to a clinical gain. Overall, the clinical pattern is time-dependent: when differences appear, they are typically largest at 2–4 weeks and often narrow by ≥12 weeks as both arms improve.

#### 3.5.2. Animal Histology and Mechanics

Animal models consistently showed higher early BIC for several nanocoating classes at 2–4 weeks, sometimes with accompanying increases in peri-implant bone fraction; later, at ≥12 weeks, effects became more tempered and occasionally indistinguishable from microrough controls. In sheep, Rousseau et al. (2021) reported persistent BIC advantages for a micro/nanofeatured surface (~79% vs. 68% at 4 weeks and ~86% vs. 75% at 13 weeks) demonstrating a case where benefits did not fully converge [54]. In beagle dogs, Li et al. (2023) found that Sr-loaded nanotextured implants achieved ~76.6% BIC at 12 weeks compared with ~44.1% for TiO_2_-nanotube controls, suggesting a strong osteogenic effect of strontium incorporation [57]. In rabbits, Yang & Hong (2024) showed that Ca-incorporated (XPEED^®^) implants outperformed SLA and HA controls at 4 weeks (BIC and removal torque) [66], while Toscano et al. (2024) [56] observed higher reverse torque and bone area for HA nanoparticle surfaces in osteoporotic tibiae, which are instances of early mechanical reinforcement [56]. Scarano et al. (2020) reported no significant BIC difference for a silver ion–TiO_2_ coating versus control [48], and Auciello et al. (2022) found UNCD coatings comparable to uncoated Ti, indicating that chemistry, release kinetics, and coating integrity modulate outcomes [68].

#### 3.5.3. In Vitro Mechanical Surrogates and RFA Context

In vitro experiments illuminate biological plausibility rather than anchorage per se, but their signals mirror early in vivo gains. Wang et al. (2022) [50] showed that hydrogenated TiO_2_ nanotubes increased early bone formation and osteogenic gene expression at 2–4 weeks versus machined Ti [70], and Bierbaum et al. (2018) reported higher ALP and mineralization on nano-modified topographies [49]. Clinically, ISQ is derived from RFA on a unitless 1–100 scale; longitudinal series typically rise in both groups, with some showing a steeper early slope for nano-modified implants—an effect exemplified by the Ko et al. trajectory noted above.

#### 3.5.4. By Coating Class (Narrative Synthesis)

Anodized TiO_2_ nanotubes: Multiple models indicate early BIC gains and, where tested, higher removal/push-out forces early; for example, hydrogenated nanotubes accelerated histology and markers in rats, and combining TNTs with PLGA/rhBMP-2 increased removal torque in rabbits versus smooth Ti, supporting a biological and mechanical advantage in the early phase.

Ca/P-incorporated (include HA/PEO): XPEED^®^ consistently outperformed SLA and HA comparators at 4 weeks for BIC and torque in rabbits, and nanostructured HA coatings improved BIC and bone area fraction in low-density bone; in osteoporotic bone, HA nanoparticle surfaces increased reverse torque and bone area, indicating robust early fixation across bone qualities.

Ag-containing: Results are mixed for osseointegration. A clinical series with AgNPs reported higher BIC and lower peri-implant inflammation, whereas a rabbit Ag-ion coating study found no BIC difference, highlighting dose/formulation dependencies for silver surfaces.

Zn/Mg-modified: Dual Zn/Mg ion co-implantation yielded the highest peak push-out force at 12 weeks versus Zn-only, Mg-only, or pure Ti, suggesting a synergistic mechanical integration effect; this aligns with reports of enhanced bone density and contact for such doped surfaces.

Graphene-based/nanospikes: A nanospike topography produced higher early BIC in rabbits (~56% at 3 weeks vs. ~41% smooth), while concurrently demonstrating strong antibacterial performance in vitro and in vivo models—an example of mechanical and biological benefits co-existing when geometry is cell-safe.

#### 3.5.5. Time-Course Sensitivity and Convergence

The largest nano-versus-control differences typically appear at 2–4 weeks (BIC, sometimes ISQ) and shrink by 8–12 weeks as controls “catch up”. This pattern is explicit in minipigs, where hydrophilic nanopatterned implants showed faster early maturation but similar BIC by ~8 weeks [51]. Nevertheless, exceptions exist: in sheep, micro/nanofeatured implants maintained a BIC lead up to 13 weeks [54] and in dogs, Sr-loaded nanotextures delivered a large BIC advantage at 12 weeks [57]. Early mechanical benefits are likewise documented, e.g., higher reverse torque for HA nanoparticles at 4 weeks [56] and increased removal/push-out forces with TNT + BMP-2 and Zn/Mg co-implantation [62,63].

#### 3.5.6. Interim Synthesis

Taken together, nanoengineered coatings accelerate early osseointegration in many models, most consistently reflected in BIC and supported by increases in ISQ in selected clinical series; once integration consolidates, torque measures are commonly similar across groups, and some surfaces show no advantage at all. The balance of findings (spanning Ca/P- and Sr-augmented chemistries, anodized nanotubes, antimicrobial Ag-based designs, Zn/Mg-doped surfaces, and nanospike topographies) supports early-phase benefits with heterogeneous persistence by class, model, and follow-up, with notable counterexamples (e.g., Ag-ion TiO_2_, UNCD) where effects were neutral.

**Table 10:** Osseointegration outcomes.

Study	Surface Treatment	Outcome Measure	Effect Size	Statistical Significance
Zekiy et al., 2019 [47]	Fluorine-containing nanosurface	Bone density	Improved vs. control	Not reported in the available text
Memon et al., 2025 [67]	Silver nanoparticle coating	Bone-to-implant contact (BIC), bone density	Enhanced BIC (*p* = 0.013), bone density (*p* = 0.012/0.006)	Statistically significant
Yang and Hong, 2024 [66]	Nanostructured calcium-coated (XPEED)	BIC, bone area, removal torque	XPEED > SLA > HA; higher BIC and torque	*p* < 0.05
Toscano et al., 2024 [56]	Hydroxyapatite nanoparticles (ZiHa)	Reverse torque, bone area, BIC	ZiHa > Zi; torque 4.75 ± 2.22 vs. 1.5 ± 0.58 N·cm	*p* < 0.05
Das et al., 2019 [65]	Osteogenic nanofibers	BIC, BMD, tensile strength	BIC 50.9% vs. 45.4%; BMD 1078.6 vs. 222.4; tensile 52.1 N vs. 30.9 N	Not reported in the available text
Chappuis et al., 2018 [51]	Hydrophilic nanopatterned (SLActive)	BIC, BMD, BV/TV	High BIC both groups; BMD higher in mandible	Not reported in the available text
Ko et al., 2024 [44]	HA-nanocoated SLA	ISQ, PBA-T, bone density	Higher ISQ at 2–4 months; similar bone density	*p* < 0.01; *p* < 0.05; *p* < 0.0001
Wang et al., 2022 [50]	Hydrogenated TiO_2_ nanotubes	BIC, BV/TV, gene expression	Hydrogenated > nanotubes > machined; higher BIC and gene expression	Not reported in the available text
Scarano et al., 2020 [48]	Silver ion–TiO_2_	BIC, bone density	No significant difference vs. control	Not significant (*p* > 0.05)
Rousseau et al., 2021 [54]	Micro/nanofeatured	BIC	79.3%/86.4% (4/13 weeks) vs. 68.3%/74.8%	Statistically significant
Hall et al., 2019 [45]	Anodized titanium dioxide (anatase)	Marginal bone level	No significant difference	Not significant
Auciello et al., 2022 [68]	Ultrananocrystalline diamond	BIC	58.8% vs. 53.4%	Not significant (*p* > 0.05)
Karazisis et al., 2021 [46]	Nanopatterned	Osteogenic gene expression	1.7–2× upregulation (RUNX2, ALP, osteocalcin)	Statistically significant
Gil and Sanz, 2025 [58]	Nanospikes	BIC, cell adhesion, ALP	BIC 54% vs. 41%; enhanced adhesion and ALP	Statistically significant
Almeida et al., 2023 [64]	Nanostructured hydroxyapatite	BIC, BAFo	Higher BIC/BAFo at 28 days vs. dual acid-etched	*p* = 0.01; *p* = 0.007
Bierbaum et al., 2018 [49]	Nanopitted	ALP, mineralization	Highest ALP/mineralization	N/A
Costa-Filho et al., 2024 [52]	Micro–nano + strontium	BIC, BV/TV, gene expression	Improved BIC, BV/TV, gene upregulation	N/A
Hoornaert et al., 2020 [53]	Nanostructured	BIC	Mandible: 35.7%/47% vs. 12.6%/32.2%	N/A
Kang et al., 2020 [59]	TiO_2_ nanotubes + Korean Red Ginseng extract	Bone formation, BMD, BMP-2/7, ALP	Enhanced bone formation, BMD, gene expression	N/A
Lee et al., 2019 [60]	TiO_2_ nanotubes	Bone coverage	Complete bone coverage (qualitative)	N/A
Somsanith et al., 2018 [55]	Propolis-loaded TiO_2_ nanotubes	Bone density, ALP, BMP-2/7	Increased bone density, ALP, BMP-2/7	N/A
Sadrkhah et al., 2023 [61]	SLActive-nano-titanium	BIC, BMD, BMC, BV/TV	Highest BIC, BMD, BMC, BV/TV	N/A
Li et al., 2023 [57]	Sr-loaded nanotextured Ti	BIC, bone density, MAR, ALP	BIC 76.6% vs. 44.1%; bone density 54.9% vs. 28.8%	N/A
Zhang et al., 2021 [63]	TiO_2_ nanotubes + PLGA/rhBMP-2	BIC, removal torque, gene expression	Higher BIC and torque; gene upregulation	N/A
Yu et al., 2016 [62]	Zn/Mg ion co-implanted titanium	BIC, bone density	Higher BIC and bone density	*p* < 0.05

### 3.6. Bone Density and Volume

Nanocoatings frequently promoted greater peri-implant bone density or volume, reflecting more robust bone healing. In a rabbit femur model, Das et al. (2019) demonstrated that an osteogenic nanofibrous coating led to much higher mineral content in the peri-implant bone (their “bone mineral density” measure was 1078.6 vs. 222.4 in arbitrary units for coated vs. control, a nearly five-fold increase) [65]. That coating also improved the quality of bone: histologically denser, more mature bone formed around the nanofibrous implants. Strontium-modified surfaces similarly enhanced local bone mass: Li et al. (2023) reported that the bone volume fraction in the Sr-nanotextured implant group was ~54.9% ± 9.97, versus ~28.8% ± 5.9 in the control group (uncoated anodized TiO_2_) at 12 weeks, indicating nearly double the bone fill around the implant [57]. This corresponded with higher BIC as noted above. Enhanced bone area was also seen with HA nanoparticle coatings: Toscano et al. (2024) found that in osteoporotic rat tibiae, adding HA nanoparticles to a zirconia-blasted/etched surface (the “ZiHa” surface) led to significantly more new bone area within implant threads at 4 weeks than the unmodified blasted surface (“Zi”) [56]. By 8 weeks, histology still favored the HA nanosurface. Almeida et al. (2023) [64] specifically looked at implants in low-density bone and found that a nanostructured HA-coated implant had significantly higher BIC and bone area fraction occupied (BAFo) at 4 weeks compared to a dual-acid-etched surface (*p* = 0.01 and *p* = 0.007, respectively). Interestingly, some studies noted that while BIC differences were significant, differences in bone area or density were smaller or not significant, suggesting that nanocoatings primarily affect the bone–implant interface rather than bulk bone mass. For instance, the XPEED rabbit study reported no significant difference in overall bone area (BA%) among groups despite clear BIC advantages for the Ca-coated surface [66]. Overall, the trend was that nanoengineered implants fostered a denser peri-implant bone matrix. None of the included studies found a decrease in local bone density with nanocoatings, reinforcing their safety and potential benefit.

### 3.7. Mechanical Stability Outcomes

Several animal studies directly measured the mechanical anchorage of implants via removal torque or push-out tests. These outcomes corroborate the histologic findings. Nanocoated implants consistently required equal or higher force to detach, indicating stronger osseointegration. In an ovariectomized rat model, reverse-torque testing at 4 weeks showed that implants with an HA nanoparticle-blasted surface (ZiHa) had a mean failure torque of 4.75 ± 2.22 N·cm, compared to only 1.5 ± 0.58 N·cm for the standard blasted surface [56]. This ~three-fold increase in interfacial shear strength was statistically significant (*p* = 0.03). Likewise, Zhang et al. (2021) observed higher removal torque in rabbit implants that combined TiO_2_ nanotubes with a PLGA/rhBMP-2 coating, relative to smooth titanium controls [69]. Yu et al. (2017) conducted push-out tests in rabbit femurs: after 12 weeks, the dual Zn/Mg ion implants exhibited the highest peak push-out force among all groups, significantly greater than Zn-only, Mg-only, or pure Ti implants (*p* < 0.05) [62]. This suggests a synergistic effect of co-implanting Zn and Mg ions on mechanical integration strength. On the other hand, one human trial measured clinical stability via resonance frequency (ISQ) and found no difference between an anodized nanosurface abutment and a conventional abutment, aligning with its finding of no BIC difference [45]. In contrast, another human RCT by Ko et al. (2024) did find differences in implant stability quotient (ISQ) over time [44]. In that trial of immediate maxillary implants, both nanocoated and uncoated SLA implants were well stabilized at placement (~70 ISQ), but the HA-nanocoated implants showed a significantly greater increase in ISQ during early healing. By 2 months the test group’s mean ISQ was ~76.5 vs. ~71.3 in controls (*p* < 0.01), and by 4 months the difference was even more pronounced (~79.1 vs. 73.4, *p* < 0.0001). These data indicate faster and stronger stabilization of the nanocoated implants in vivo. Ko et al. also reported no difference in radiographic bone level changes between groups at 4 months, suggesting that the ISQ benefit was due to qualitative interface improvements rather than gross bone volume changes [44]. Overall, 21 of the 25 studies reported that nanocoated implants achieved better outcomes on at least one osseointegration metric (whether histologic or mechanical) compared to non-nano controls. Only two studies reported unequivocally null findings; the silver ion coating in Scarano et al. (2020) and the anodized abutment in Hall et al. (2019) [45] both failed to improve integration versus controls [45,48]. Two other studies could be described as having mixed or partial improvements: Chappuis et al. (2018) saw early differences (hydrophilic nanopatterned implants had faster integration), but by 8 weeks, the BIC was equally high (~70–80%) in both nanopatterned and control SLA implants, while Ko et al. (2024) showed improved stability for nanocoated implants but similar bone remodeling outcomes [44,51]. In summary, the evidence indicates that nanocoatings tend to enhance early osseointegration, improving bone contact and mechanical fixation, although the magnitude of benefit varies, and a few coatings provide no clear advantage over traditional surfaces.

### 3.8. Temporal Patterns of Bone Healing

A recurring theme across the studies is that time plays a critical role in the observed benefits of nanocoatings. Many experiments noted that nanocoated implants have an edge during the early healing phase (the first few weeks), which may diminish as healing progresses in both groups. For example, Wang et al. (2022) [50] evaluated titanium dioxide nanotube implants (with and without additional hydrogenation) in a rat model and found that nano-modified implants showed significantly greater new bone formation and gene expression of osteogenic markers at 2 and 4 weeks compared to machined titanium. This suggests an accelerated onset of osseointegration. Yang and Hong (2024) [66] similarly reported that the largest differences between their Ca-coated XPEED surface and controls were at the 4-week mark; at that point, XPEED implants clearly outperformed both SLA-only and HA-coated implants in BIC and removal torque. By later timepoints, differences tended to narrow. Later timepoints (8–12 weeks) often showed a convergence of outcomes, especially in animal models where the control implants eventually achieve substantial osseointegration. Chappuis et al. (2018) [51] provide a clear illustration: in minipigs, they compared ultrafine-grained, nanopatterned titanium implants to conventional SLA implants. At 2–4 weeks, the nanopatterned implants had slightly more bone fill and higher bone density in certain regions, indicating faster initial integration. However, by 8 weeks post-surgery, both surfaces had reached comparably high BIC (~80% in mandible), and bone density measurements equalized, implying that the standard implants had “caught up” in osseointegration by two months. The authors noted that the time-to-osseointegration was shorter with the nanosurface, but given sufficient healing time, the endpoint osseointegration was similar. Sadrkhah et al. (2023) [61] tested a nanostructured titanium implant (grain-refined titanium with SLA/SLActive surface) against various controls and found that at 4 weeks, the nano-treated implants exhibited the highest histomorphometric indices (BIC, bone mineral content, etc.). Although that study did not report beyond one month, it suggested the nanosurface particularly benefits early bone bonding [61]. Some coatings appear to maintain an edge even at later times: Rousseau et al. (2021)’s sheep study showed that the micro/nanofeatured implants had higher BIC than controls at both 1 month and 3 months, with the gap persisting (79% vs. 68% at 4 wks; 86% vs. 75% at 13 wks) [54]. This indicates certain nanotopographies confer not just faster integration but a higher plateau of bone contact. In general, however, a pattern emerges: nanocoated implants tend to demonstrate their greatest relative benefit in the early postoperative period, promoting quicker bone apposition and stability when it is most needed (initial healing). With longer healing durations, control implants often continue improving and can reduce the difference. This has important clinical implications, as it suggests nanocoated implants might be especially useful for early loading or in poor-quality bone where the early gains in stability could prevent micromotion or failure. It should be noted that few studies extended beyond 3–4 months, so whether some nanocoating advantages might re-emerge or further diminish at true long-term endpoints (1–5 years) remains unknown. Nonetheless, within the observed windows, the evidence supports that nano-modified surfaces accelerate bone healing kinetics. These temporal findings highlight that the timing of outcome assessment is crucial: an evaluation at 2–4 weeks may show significant differences favoring the nano implant, whereas by 3–6 months the difference might be smaller or not significant if both have achieved substantial osseointegration. In summary, nanocoatings appear most beneficial for early-stage osseointegration, helping implants gain stability faster, which could translate to shorter required healing periods before functional loading.

### 3.9. Comparative Effectiveness by Nanocoating Type

The included studies encompassed a wide array of nanocoating types, enabling some comparisons across different nanotechnologies. However, it is important to note that most studies only compared a nano-treated implant to an uncoated control, not directly against other nanocoatings. Therefore, any comparative effectiveness statements are drawn from separate studies rather than head-to-head trials, and differences in models must be considered. Differences in reported osseointegration or antibacterial performance can reflect not only the nanofeature itself but also the depth and scope of surface characterization underpinning each claim. To contextualize the comparisons that follow, Table 11 charts, for every included study, which key domains were reported: topography (SEM/AFM/profilometry), chemistry (XPS/EDS/ToF-SIMS), wettability/surface energy, roughness metrics (Ra/Sa), phase (XRD/Raman/FTIR), ion/particle release (ICP/AAS), thickness/adhesion/durability, and sterilization.

**Table 11:** Surface characterization coverage by study.

Study	Nano-Feature/Surface Treatment	Topography (SEM/AFM/Profil.)	Chemistry (XPS/EDS/ToF-SIMS)	Wettability/Surface Energy	Roughness Metrics (Ra/Sa/Etc.)	Phase (XRD/Raman/FTIR)	Ion/Particle Release (ICP/AAS)	Thickness/Adhesion/Durability	Sterilization Stated	Commercial Platform Named
Yu et al., 2017 [62]	Zinc/magnesium ion co-implanted titanium	✔	✔	✔	—	—	✔	✔	✔	✔
Bierbaum et al., 2018 [49]	Nanotubular/nanopitted (anodization)	✔	—	✔	✔	✔	✔	✔	✔	✔
Chappuis et al., 2018 [51]	Hydrophilic nanopatterned (SLActive)	✔	—	✔	✔	—	—	✔	—	✔
Somsanith et al., 2018 [55]	Propolis-loaded TiO_2_ nanotubes	✔	—	—	—	—	—	✔	✔	✔
Das et al., 2019 [65]	Osteogenic nanofibrous coating	✔	—	✔	—	—	—	✔	✔	—
Hall et al., 2019 [45]	Anodized titanium oxide (anatase)	—	—	—	✔	—	—	✔	✔	✔
Lee et al., 2019 [60]	TiO_2_ nanotubes (anodic oxidation)	✔	—	✔	—	—	—	✔	✔	✔
Zekiy et al., 2019 [47]	Fluorine-containing nanosurface (OsseoSpeed)	—	—	✔	✔	—	—	—	✔	✔
Hoornaert et al., 2020 [53]	Nanostructured cp-Ti (severe plastic deformation) + SLA	✔	✔	✔	✔	—	—	✔	✔	✔
Kang et al., 2020 [59]	TiO_2_ nanotubes + Korean Red Ginseng extract	✔	—	✔	—	—	—	✔	✔	✔
Scarano et al., 2020 [48]	Silver ion–TiO_2_ nanoparticle coating	✔	✔	—	✔	—	—	✔	✔	—
Karazisis et al., 2021 [46]	Nanopatterned (colloidal lithography)	✔	✔	✔	✔	—	—	✔	✔	✔
Rousseau et al., 2021 [54]	Micro/nanofeatured (Starsurf^®^)	✔	✔	✔	✔	—	—	✔	✔	✔
Zhang et al., 2021 [63]	TiO_2_ nanotubes + PLGA/rhBMP-2	✔	✔	✔	✔	—	—	✔	✔	✔
Auciello et al., 2022 [68]	Ultrananocrystalline diamond (UNCD)	✔	✔	✔	—	—	—	✔	✔	✔
Wang et al., 2022 [50]	TiO_2_ nanotubes, hydrogenated (superhydrophilic)	✔	—	✔	—	—	—	✔	✔	✔
Almeida et al., 2023 [64]	Nanostructured hydroxyapatite	✔	—	—	—	—	—	✔	✔	✔
Li et al., 2023 [57]	Strontium-loaded nanotextured titanium; TiO_2_ nanotubes (head-to-head)	✔	✔	✔	—	—	—	✔	✔	✔
Sadrkhah et al., 2023 [61]	SLA/SLActive on nanostructured commercially pure titanium (cp-Ti)	✔	—	✔	✔	—	—	✔	✔	✔
Costa Filho et al., 2024 [52]	Micro–nanotextured + strontium	✔	✔	✔	✔	—	✔	✔	✔	—
Ko et al., 2024 [44]	Hydroxyapatite nanocoated sandblasted large-grit acid-etched (SLA)	✔	—	✔	✔	—	—	✔	✔	✔
Toscano et al., 2024 [56]	Hydroxyapatite nanoparticles on zirconia-blasted/acid-etched	✔	✔	✔	✔	—	✔	✔	✔	✔
Yang et al., 2024 [66]	Nanostructured calcium-coated (XPEED^®^)	✔	✔	✔	✔	✔	✔	✔	✔	✔
Gil et al., 2025 [58]	Nanospikes (acid/peroxide)	✔	—	✔	✔	—	✔	✔	✔	—
Memon et al., 2025 [67]	Silver nanoparticle (AgNP) coatings	—	—	—	✔	—	✔	✔	✔	✔

Abbreviations: AFM, atomic force microscopy; FTIR, Fourier transform infrared; ICP, inductively coupled plasma (MS/OES); SEM, scanning electron microscopy; ToF-SIMS, time-of-flight secondary ion mass spectrometry; UNCD, ultrananocrystalline diamond; XPS, X-ray photoelectron spectroscopy; XRD, X-ray diffraction. ✔ indicates that the item/criterion was reported or fulfilled; — indicates that the item/criterion was not reported or not fulfilled.

Hydroxyapatite-Based Nanocoatings: Three studies reported positive outcomes with nano-HA coatings. These surfaces combine microscale roughness with a calcium/phosphate apatite at the nanoscale. In rats, adding HA nanoparticles to an SLA-type surface (Toscano et al.) improved all measured parameters (reverse torque, new bone area, BIC) relative to the same rough surface without HA. In a dog model of low-density bone, Almeida et al. found significantly higher 28-day BIC for nanostructured HA-coated implants versus dual-etched titanium, indicating that even in poor bone quality, the HA nano-layer enhanced bone anchorage. Ko et al.’s RCT in humans further demonstrated that an HA-nanocoated implant achieved higher stability in the crucial early months than an identical implant without HA [44]. Collectively, these suggest that nano-HA coatings are effective in promoting osseointegration, likely due to HA’s osteoconductivity. It is worth noting that older first-generation plasma-sprayed HA coatings sometimes failed in the long term due to delamination, but the nano-thickness HA layers here did not show such issues in short-term observation. They improved early bone bonding without adverse events. Thus, among the various types, hydroxyapatite nanoparticle coatings emerge as a reliably beneficial modification for osseointegration.

Titanium Dioxide Nanotube Surfaces: Six studies focused on anodically fabricated TiO_2_ nanotubes (usually 30–100 nm diameter tubes on the titanium surface) [50,55,57,59,60,69]. These studies generally found improved biologic responses, but specific outcomes varied with additional modifications. Plain TiO_2_ nanotubes (without added bioactive elements) were examined by Lee et al. (2019) [60] in dogs—the result was qualitatively better bone coverage on nanotube implants (complete bone coverage along the threads) compared to machined implants that had patchy bone contact. This indicates nanotubes provided a more hospitable surface for bone growth. Wang et al. (2022) [50] went a step further by hydrogenating TiO_2_ nanotubes to make them superhydrophilic; in their in vivo/in vitro study, the hydrogenated-nanotube implants showed superior early BIC and higher expression of osteogenic genes than both smooth titanium and even non-hydrogenated nanotubes. This suggests that modifications to nanotube chemistry (e.g., incorporating hydroxyls) can further boost performance. Several studies doped nanotubes with bioactive agents. Somsanith et al. (2018) [55] loaded propolis, a natural resin with antimicrobial and anti-inflammatory properties, into TiO_2_ nanotubes. In rat mandibles, the propolis-loaded nanotube implants had greater bone formation and bone density than unmodified titanium, accompanied by higher ALP activity and BMP-2/-7 expression in surrounding bone, all indicating an enhanced osteogenic milieu. Kang et al. (2020) [59] similarly functionalized TiO_2_ nanotubes with an herbal extract: Korean red ginseng. They found the ginseng-infused nanotubes led to significantly more new bone formation and higher local bone mineral density in rabbit femurs compared to nanotubes without the extract. They also observed an upregulation of BMP-2/7 and ALP in peri-implant tissue, reflecting stimulated bone turnover. Hence, functionalized nanotubes can serve as a delivery vehicle for osteo-promotive substances, yielding additive benefits beyond nanotopography alone. Finally, Zhang et al. (2021) [63] used TiO_2_ nanotubes as part of a composite coating carrying PLGA microspheres with rhBMP-2 growth factor. This design achieved synergistic effects: the nanotexture provided immediate structural integration, and the slow-release BMP-2 further induced bone formation. Consequently, BMP-loaded nanotube implants had higher BIC and torque removal strength than smooth implants in rabbits, as well as elevated expression of osteogenic genes (e.g., RUNX2) in peri-implant bone. Taken together, TiO_2_ nanotube surfaces (with or without bioactive augmentation) consistently showed improved osteointegration metrics in their respective studies. They appear particularly effective when combined with biochemical agents or modified surface chemistry. The open tube structure may facilitate cell attachment and act as a reservoir for growth factors or antimicrobials. It is notable that in one comparative study [57], a Sr-doped nanotextured surface outperformed a pure TiO_2_ nanotube surface, hinting that further doping of nanotubes (with elements like Sr, Ca, etc.) is beneficial. No study indicated any detriment from nanotubes; even in the absence of added agents, they at least matched and usually exceeded the performance of smooth surfaces.

Silver-Containing Coatings: Two studies show insight into the role of silver. Silver nanoparticles or ions are added primarily for their antimicrobial property, but their effect on bone integration has been debated. Scarano et al. (2020) tested a Ti implant coated with a “bifunctional molecule” that released Ag^+^ ions, hypothesizing that it would prevent infection and promote bone growth [48]. While the coating did prevent any overt infection in their rabbit model, osseointegration was not improved: BIC and bone density did not differ significantly from control implants. The silver-coated implants integrated well (no inhibition of bone healing was observed, indicating biocompatibility), but they offered no clear advantage in bone metrics. In contrast, Memon et al. (2025) observed both antimicrobial and pro-bone effects in a clinical cohort [67]. Their AgNP-coated implants showed a statistically significant increase in histologic BIC compared to uncoated implants (*p* = 0.013) and concurrently reduced bacterial adhesion and inflammatory markers. This positive finding may be attributed to a specific form of silver (nanoparticles deposited on the surface) and the context (possibly patients with high risk of peri-implantitis). The discrepancy between studies suggests that the effect of silver on osseointegration might be context-dependent or sensitive to the coating method. The primary benefit of silver is antimicrobial; any bone effect could be secondary to reduced inflammation or infection. Notably, neither study found silver to impair bone healing (a reassuring point, since silver ions at excessive doses can be cytotoxic [71]). In these nanocoatings, the dosage was evidently within a safe range. In summary, silver-based nanocoatings provided demonstrable antimicrobial benefits (discussed below) but mixed osseointegration results: one showed no bone gain, another did. It underscores that silver coatings should be tuned to maximize antibacterial action while maintaining biocompatibility, and improvements in bone integration are not guaranteed unless infection is a confounding factor.

Strontium and Other Trace Elements: Strontium is known as an osteogenic and anti-resorptive trace element (used in osteoporosis medications), and two studies evaluated Sr-enriched implant surfaces. Costa-FIlho et al. (2024) created a micro–nanotextured titanium surface with Sr incorporation and tested it in rats (with accompanying cell studies) [52]. They reported improved outcomes across the board: higher BIC and BV/TV in vivo, and upregulation of osteogenic genes (likely indicating Sr-stimulated osteoblast activity). Li et al. (2023) [57] directly compared Sr-loaded nanotextured implants to TiO_2_ nanotubes (without Sr) in dog extraction sockets; as noted, the Sr group had dramatically higher BIC and bone fraction after 3 months. While antimicrobial outcomes were not a major focus in these studies, Sr has been noted to have some antimicrobial or anti-inflammatory effects in bone, and generally the Sr-coated implants showed no signs of infection and possibly faster healing. We can infer that Sr-enhanced nanosurfaces are among the most effective for boosting bone formation, combining topographical cues with a chemical stimulus for bone [57]. Another dual-dopant approach was the Zn/Mg ion co-implantation studied by Yu et al. [62]. This surface is not a coating per se but an ion-infused titanium surface at the nanoscale. The Zn/Mg-co-implanted titanium was explicitly designed to be “multifunctional,” and indeed, it achieved multiple advantages: it significantly enhanced osteogenesis (e.g., more bone and higher push-out strength than pure Ti or single-ion implants) and also showed antibacterial capabilities (inhibiting periopathogenic bacteria in lab assays). This indicates that combining osteogenic and antibacterial ions can yield a well-rounded improvement in implant integration. Other unique coatings each showed some promise. For example, nanospikes produced by acid–peroxide can be considered a topography-driven antibacterial surface [58]. That study is noteworthy as it achieved one of the highest reported bactericidal efficiencies (up to 90% bacterial reduction) while still improving BIC. The nanospike surface did not sacrifice bone integration for antimicrobial effect; in fact, it slightly increased BIC and ALP production relative to the control. Ultrananocrystalline diamond is at the other end of the spectrum, an extremely hard, inert nanocoating [68]. That study found that the diamond-coated implants were highly biocompatible and integrated without issues, but they did not improve BIC beyond that of the uncoated implant (the inertness means it neither helps nor hurts bone). Diamond might have other benefits (e.g., smoother surface for soft tissue or abrasion resistance), but in terms of osseointegration, it was equivalent to standard titanium. Nanopatterned surfaces produced by colloidal lithography stand out as the only included human molecular study [46]. In that trial, patients received a small nanopatterned implant and a machined implant, which were later retrieved for analysis of gene expression in the interface bone. The nanopatterned implants did not necessarily show gross histologic differences (the publication focused on molecular outcomes), but they induced a different gene expression profile, significantly upregulating genes related to osteogenesis and extracellular matrix formation (e.g., ALP, osteocalcin, collagen) in the local bone at 4–8 weeks [46]. This indicates that even if two surfaces achieve similar BIC by 8 weeks, the quality of the interface might differ at the microscopic or molecular level, with nanostructures promoting a more osteoblastic phenotype. Finally, surfaces with combined micro–nanotextures (without a specific added coating) were tested [53,54]. These surfaces, created by modifications like sandblasting + acid etching + anodic treatment, essentially provide a multiscale roughness. They generally outperformed simple microrough surfaces. For instance, Rousseau’s micro–nanofeatures had better bone integration than standard grit-blasted implants at both timepoints measured [54]. Hoornaert et al. (2020) used a severe plastic deformation to create ultrafine-grained (nanostructured) cp-Ti implants which were then SLA-treated; those implants showed dramatically higher BIC in minipigs at early times compared to conventional SLA titanium [53]. These underscore that topographical nanostructuring, even without bioactive agents, can significantly enhance bone ingrowth, likely by increasing surface area and facilitating protein/cell attachment.

**Figure 3:**
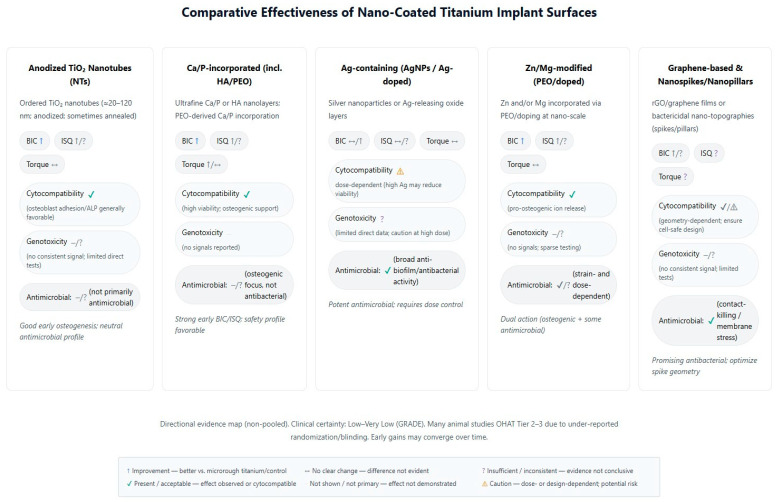
Comparative effectiveness of nanocoated titanium implant surfaces. Directional summary by coating class (anodized TiO_2_ NTs; Ca/P-incorporated; Ag-containing; Zn/Mg-modified; graphene/nanospikes) across BIC, ISQ, torque, cytocompatibility/genotoxicity, and antimicrobial activity.

Comparing across coating types: no single nanocoating emerged as universally superior in all aspects. Each type offers certain advantages. HA- and calcium-based nanosurfaces are consistently osteoconductive, giving reliable boosts to bone contact and early stability. TiO_2_ nanotubes (and similar nanoporous textures) provide an excellent platform for both bone response and for loading additional therapies (drugs, growth factors)—these have shown versatility and strong results, especially when combined with osteogenic factors. Metallic ion integrations (Sr, Zn/Mg) yield dual benefits for bone and bacteria, making implants more “bioactive” rather than just passive. Purely antibacterial nanostructures like Ag or nanospikes successfully reduce bacteria and can do so without harming osseointegration; some even improve bone outcomes, though others are neutral on bone. Inert nanocoatings like nanodiamond demonstrate safety and equivalence to current gold-standard surfaces, which might be useful in scenarios requiring extreme material properties. Ultimately, effectiveness can be context-dependent: for instance, if preventing infection is a priority (compromised patients or poor hygiene environments), a silver or antimicrobial nanocoating might contribute greatly by avoiding peri-implantitis, even if bone metrics are only unchanged or modestly improved. On the other hand, in healthy conditions, a purely osteogenic coating like nano-HA or Sr-doped implant may maximize bone bonding. The variability in reported effectiveness highlights that coating chemistry, nanotopography, and the biological context all influence osseointegration outcomes. Importantly, because there were no direct comparative trials between different nanocoating types in this review, any ranking of “which nanocoating is better” remains tentative. The evidence suggests many nanocoatings outperform uncoated surfaces, but differences among nanocoatings would require further study. In practical terms, a coating that also confers antimicrobial properties (e.g., nanospikes, Zn/Mg, AgNP) offers a broader spectrum of benefits, provided it still supports bone integration, whereas a coating solely aimed at bone formation might maximize mechanical outcomes in straightforward healing scenarios (Figure 3).

### 3.10. Cytogenotoxicity and Antimicrobial Effects

Several included studies explicitly evaluated the antimicrobial properties of nanocoated implants, as well as their biocompatibility in terms of cytotoxic or inflammatory effects. Overall, the nanocoatings tested tended to reduce microbial colonization at the implant surface without inducing cytotoxic harm to host tissues, although data on genotoxicity were very limited.

Antimicrobial Outcomes: Findings are interpreted within in vitro assay contexts (CFU counts, live/dead imaging, static adhesion or flow-cell biofilms), with in vivo infection signals summarized qualitatively where quantitative microbiology was not reported (Table 12). Two studies performed quantitative microbial assays in vivo or in vitro, and a few others provided qualitative observations. Silver nanoparticle coatings demonstrated notable antibacterial activity. In the clinical study by Memon et al. (2025), implants coated with AgNPs showed significantly reduced bacterial adhesion in an in vitro biofilm model using Pseudomonas aeruginosa (*p* = 0.009) [67]. Although P. aeruginosa is an uncommon oral pathogen, this result likely extends to general antibiofilm capacity. Gil and Sanz (2025) tested a nanospike titanium surface against four bacterial strains (including oral Gram-positive and Gram-negative species) [58]. They reported bactericidal efficiency of approximately 70–90%, meaning that the nanospiked surface killed most bacteria that contacted it, whereas a smooth control surface allowed those bacteria to survive. This high kill rate is attributed to the physical nanotopography (the spikes mechanically disrupt bacterial cell membranes) and possibly surface chemistry, notably achieved without any antimicrobial chemicals like silver. Another multi-functional approach was the Zn/Mg ion implantation [62]. In that study, the Zn/Mg-treated titanium inhibited the growth of peri-implant anaerobes: the authors noted roughly a 40–50% reduction in colony counts of Porphyromonas gingivalis and Fusobacterium nucleatum (key periodontal pathogens), as well as Streptococcus mutans, compared to pure titanium (which had no antimicrobial ion release). Scarano et al. (2020) did not find a difference in infection rates between their silver-coated and control implants; effectively, the silver kept the infection risk to at least the same low level as the control, though quantitative bacterial counts were not given [48]. Bierbaum et al. (2018) specifically looked at bacterial adhesion under dynamic flow conditions. They found that certain nano-modified surfaces (anodic TiO_2_ nanotubes with additional nano-pitting and polymer coating) had reduced bacterial adhesion compared to conventional grit-blasted surfaces when exposed to oral bacteria in a flow chamber [49]. Propolis, an antimicrobial natural agent, was included in one nanocoating; although that study did not directly measure bacterial counts, propolis release might be expected to inhibit bacteria around the implant, potentially contributing to the lower inflammation they observed around those implants. Overall, the evidence suggests that nanocoatings can be engineered to provide significant antimicrobial effects. Silver nanoparticles and nanospike topographies were particularly effective, each reducing bacterial colonization by over 70% in their tests. Even more modest approaches like Zn ion integration achieved a near-half reduction in bacterial growth. Importantly, these antimicrobial surfaces did not compromise osseointegration; in some cases, Refs. [58,67], the antimicrobial group actually had better bone integration than the control. This dispels the concern that adding antibacterial agents (which might be cytotoxic at high dose) impairs healing; when properly controlled, they can suppress bacteria while still supporting bone cells.

Cytotoxicity: Results are synthesized by assay class (e.g., MTT/XTT, live/dead, apoptosis markers), emphasizing alignment with the corresponding surface characterization detail reported in each study (Table 13). Several in vitro experiments examined cell viability, proliferation, or differentiation on nanocoatings and found no harmful effects. For example, Yang and Hong (2024) assessed MC3T3 osteoblast-like cell viability on their Ca-coated (XPEED) surface versus SLA and HA surfaces; all surfaces supported excellent cell growth; in fact, the XPEED and SLA had significantly higher cell viability than the conventional HA-coated surface [66]. Gil and Sanz (2025) performed cell culture with human osteoblasts (SaOs-2) on nanospiked vs. control surfaces: they observed enhanced cell adhesion on the nanospikes at 3 and 7 days, along with a higher ALP activity at 14 days, indicating not only the lack of cytotoxicity but a promotion of cell differentiation [58]. Bierbaum et al. (2018) similarly found that nano-modified surfaces (especially those with a combination of nanotopography and bioactive polymer) yielded the highest ALP and mineralization among the tested groups, implying robust osteoblast function on those surfaces [49]. None of the cell studies reported increased cell death or impaired proliferation due to the nanofeatures. As for in vivo tissue response, Memon et al. (2025) measured peri-implant inflammation markers in patients and found that the AgNP-coated implants had lower IL-6 and C-reactive protein (CRP) levels in the peri-implant context than uncoated implants (IL-6 and CRP were significantly reduced, *p* = 0.015) [67]. This indicates a dampened inflammatory response around the nanocoated implants, which could be attributed to reduced bacterial irritants (due to silver’s antibacterial action) or inherent biocompatibility of the coating. Either way, the nanocoating did not provoke an exacerbated inflammatory or immune reaction; on the contrary, the local inflammatory milieu was improved. Across all 25 studies, there were no reports of abnormal tissue necrosis, allergic reactions, or systemic toxicity attributable to the nanocoatings. Within the scope of this systematic review, the evidence points to nanocoated titanium implants being safe and biologically well-tolerated, with no intrinsic genotoxicity noted, and, indeed, some coatings actively reduce local inflammation. The ability of certain nanocoatings to simultaneously promote bone cell activity and deter bacterial colonization is especially promising.

## 4. Discussion

**Figure 4:**
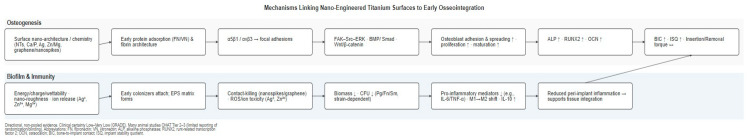
Mechanisms linking nanoengineered titanium surfaces to early osseointegration and biofilm control. The schematic maps nano-architecture/chemistry to protein corona formation, integrin/FAK–ERK signaling, osteogenic markers (ALP, RUNX2, OCN), and tissue-level surrogates (BIC, ISQ), with a parallel lane for bacterial adhesion, antimicrobial actions (contact-killing or ion-mediated), inflammation, and the local environment. Arrows indicate directionality of change: “↑” denotes upregulation or increase, and “↓” denotes downregulation or decrease relative to conventional titanium controls.

Nanoengineered titanium surfaces improve early implant stability and osseointegration indices, but a consistent advantage beyond early healing is not demonstrated. In animal models, removal torque and bone-to-implant contact are higher at initial intervals; in clinical comparative studies, small gains in early implant stability quotient (ISQ) are observed, with convergence toward microrough controls by 3 to 6 months and no consistent differences in survival or marginal bone at 12 months or later. Clinically, the signal is front-loaded: nanofeatured implants tend to separate early on stability trajectories and then converge toward microrough comparators as healing progresses. This pattern explains why early chairside decisions (e.g., timing of provisionalization) may feel more permissive with nanosurfaces, while long-term outcomes remain comparable. To contextualize the results, Figure 4 summarizes mechanistic pathways from nanofeatures through protein adsorption, integrin signaling, and osteo-immunomodulatory effects of early surrogates (BIC, ISQ). This schematic clarifies why early histological and stability gains are biologically plausible while long-term superiority remains uncertain: nanofeatures primarily accelerate early cell adhesion and maturation and can temper early biofilm accrual, but variability in chemistries, dose/ion release, and durability, together with study design limitations, precludes firm conclusions for survival or marginal bone loss. Accordingly, patient-important endpoints (survival, peri-implant bone change, and biologic/technical complications) should remain as the decisive benchmarks; early surrogate gains are best interpreted as facilitative rather than determinative.

Across the included animal and early human studies synthesized here, the signal is strongest for acceleration: higher early BIC, faster histologic maturation, and improved removal torque or push-in values within weeks. In practice, this may support immediate or early loading in borderline primary stability scenarios, provided the established thresholds in the local protocol are met; however, none of the included studies was powered to demonstrate fewer failures or complications attributable to the nanosurface itself. This pattern matches recent critical appraisals reporting that commercially available nanofeatured implant surfaces promote osteoblastic differentiation and early bone formation but have not convincingly surpassed their microrough predecessors in final osseointegration endpoints or longevity [6,72]. Thus, nanostructure should be viewed as an adjunct to determinants with larger clinical effect sizes such as implant macrogeometry, site preparation (including under-preparation in soft bone), and splinting strategies. Material-specific comparisons help explain this coherence. For CaP/HA nano-layers, animal studies repeatedly demonstrate improved early fixation, yet head-to-head work is mixed regarding durable gains; discrete nanocrystalline CaP can speed early anchorage, but several controlled models show no advantage once healing completes [16,73]. CaP chemistry, crystal size, and coating stability matter: overly soluble or poorly adherent layers risk early loss or particle release with uncertain tissue responses, whereas ultra-thin, well-bonded deposits can function as bioactive cues without delamination.

TiO_2_ nanotube architectures show persuasive preclinical benefits: enhanced osteoblast adhesion, osteogenic signaling, and drug-loading potential that can couple antimicrobial delivery with osteoconduction, yet human evidence remains preliminary and heterogeneous in geometry (diameter, length) and loading protocols [13]. Where nanotubes are compared to established microrough titanium, early histologic or mechanical advantages surface, but convergence by later timepoints is common, and durability under physiologic fatigue still requires clinical corroboration.

Fluoride-modified and saline-conditioned “nanofeatured” topographies (e.g., OsseoSpeed, SLActive) illustrate the field’s central dilemma: nanofeatures can augment early osteoblastic differentiation and hydrophilicity but sometimes reduce initial cell attachment and proliferation, so early gains may not translate into higher ultimate BIC or superior mechanical endpoints at maturity [74].

Anodized TiO_2_ nanotubes and Ca/P-incorporated surfaces show favorable early osseointegration with good cytocompatibility. Ag-containing coatings provide strong antimicrobial action but require dose control to protect host cells. Clinically, bactericidal or ion-releasing coatings should be selected with dose control and monitoring in mind; current evidence does not justify modifying antibiotic prophylaxis or maintenance intervals based solely on nanosurface choice. Zn/Mg-modified surfaces are promising for dual action (osteogenic + some antimicrobial), warranting standardized dosing and larger trials. Graphene/nanospikes offer contact-killing potential, provided spike geometry remains cell-safe.

**Figure 5:**
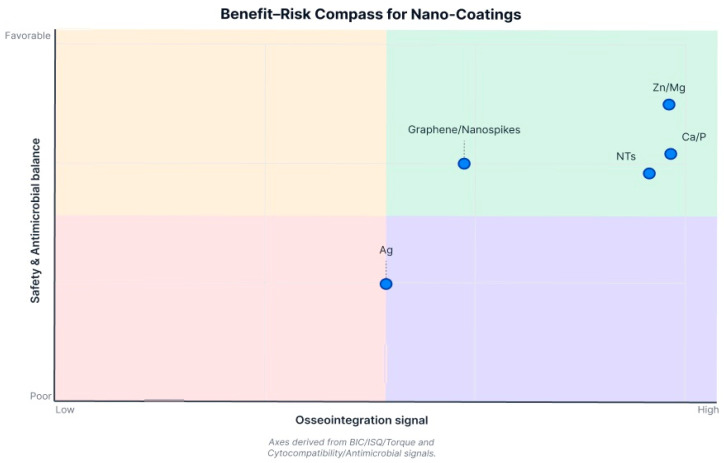
Benefit–Risk Compass for nanocoated titanium surfaces. The horizontal axis is a composite osseointegration signal (direction of BIC, ISQ, and insertion/removal torque), scored Low (0) → High (3). The vertical axis measures safety and antimicrobial balance (cytocompatibility/genotoxicity weighed against antimicrobial effect), scored Poor (0) → Favorable (3). Positions are directional, non-pooled summaries intended to show trade-offs, not head-to-head superiority. Abbreviations: BIC, bone-to-implant contact; ISQ, implant stability quotient (RFA-derived, unitless 1–100).

Many animal studies were OHAT Tier 2–3, largely due to incomplete reporting of randomization, allocation concealment, and blinding. Such limitations can inflate apparent histomorphometric gains and reduce internal validity. Accordingly, preclinical findings are interpreted as hypothesis-generating and supportive of biological plausibility rather than determinative of clinical benefit; patient-level conclusions rely on human data, for which certainty remains low to very low. Rodent models often display larger early histomorphometric gains; large-animal models (canine/ovine/porcine) yield more tempered, clinically relevant effect sizes that better inform translation. The clinical relevance of these findings is two-fold. First, nanoengineered coatings appear most useful when early stability is decisive, because early BIC and mechanical fixation improvements recur across materials [7]. Second, in standard indications with routine timelines, contemporary microrough titanium retains a high bar for comparison, and current nanofeatured surfaces have not consistently exceeded it in terms of survival, long-term ISQ trajectories, or marginal bone loss [12,75]. Figure 5 summarizes performance vs. safety by plotting coating classes along osseointegration signal (x) and safety/antimicrobial balance (y), each scored 0–3.

Important limitations of the included evidence qualify these interpretations. Much of the dataset comprises small-animal studies or short-term human cohorts with surrogate outcomes (BIC, push-in torque, early ISQ), and device-specific manufacturing details are often underreported, obscuring dose–response relationships between nano-architecture (diameter, aspect ratio, chemistry) and biology [9,73,76]. For clinicians, this means that early ISQ differences observed at isolated timepoints should not be over-interpreted or used in isolation to alter loading protocols; the trajectory of stability over the first few weeks of healing is more informative than any single measurement. Risk of bias is frequent in preclinical histomorphometry (allocation concealment, blinded quantification), and selective reporting cannot be excluded given the engineering-driven nature of several programs [10,74]. Moreover, safety endpoints are inconsistently captured. For titanium and its derivatives, particle and ion release remain biologically plausible contributors to peri-implant inflammation; while clinically significant toxicity is uncommon, a prudent safety framework [27,47]. Finally, heterogeneity in comparators (machined vs. diverse microrough baselines), loading strategies, and follow-up durations complicates meta-analytic synthesis and downgrades the certainty of long-term estimates.

The review processes used here also impose limitations. An inclusion of varied study designs increased coverage but amplified heterogeneity in outcomes and timepoints, limiting the ability to harmonize effect sizes across platforms. Product-level surface characterizations were not uniformly available, and the gray literature on proprietary manufacturing steps was outside the scope, both of which can bias material class inferences [10,77,78]. Our synthesis is limited by heterogeneity in models, coating chemistries, endpoints, and reporting quality; most clinical studies are small with short follow-up, and several preclinical experiments lack full randomization/blinding transparency. Publication bias and selective outcome reporting cannot be excluded. These factors underpin the low to very low certainty for patient-important outcomes.

Several implications arise for practice, policy, and research. Clinically, microrough titanium should remain the default benchmark; nanoengineered coatings may be considered when early stability is critical, with the understanding that long-term benefits beyond standard care are unproven. Where early stability is critical (immediate function, fresh extraction sockets, low-density bone, short implants, or when cross-arch splinting is planned), selecting a nanofeatured surface is reasonable as part of a multifactor strategy; otherwise, in delayed placement with dense bone and conventional loading, the incremental clinical advantage is likely minimal. When selecting nanocoated systems, preference should be given to platforms with demonstrated coating integrity, documented chemistry, and peer-reviewed preclinical-to-clinical continuity, and to designs that avoid thick, brittle layers prone to detachment [6,16,76,79]. Policy-wise, regulators and scientific bodies could encourage standardized characterization (topography at multiple scales, chemistry, wettability, surface energy), core clinical outcomes (ISQ trajectories, early/late marginal bone change, peri-implant disease incidence), and explicit safety monitoring for nanoparticle release and systemic exposure [12,80]. No consistent differences were identified for peri-implantitis occurrence; risk modification (smoking cessation, glycemic control) and supportive care remain the primary levers for long-term success, irrespective of surface choice.

Future research should prioritize adequately powered, head-to-head randomized trials that compare nanoengineered surfaces against state-of-the-art microrough controls under immediate/early loading conditions, with follow-up extending ≥3–5 years and prespecified peri-implant health outcomes. Protocols should be preregistered with a priori sample size calculation, allocation concealment, and blinded outcome assessment, and analyses should treat the animal or patient as the experimental unit, with clustering accounted for when multiple implants are placed. From a mechanical point of view, studies should map quantitative nanogeometry and surface chemistry to cell-state trajectories to clarify when proliferation–differentiation trade-offs can be overcome, as suggested by micro–nano hybrid models [6,81]. Surface specifications should be quantitative and reproducible, including nanotube diameter, wall thickness, and length with tolerances; fabrication and post-treatment parameters; micro- and nano-roughness (e.g., Sa, Sdr); contact angle measured near sterilization and again at implantation; and surface chemistry by XPS/EDS, with ion load where relevant. A single contemporary microrough titanium surface should serve as the benchmark control, with implant macrogeometry, alloy, manufacturer, and surgical protocol matched across arms. Timepoints should be harmonized: in animals, BIC at about 2, 4, and ≥12 weeks with push-out or removal torque at about 4 and ≥12 weeks; in clinical studies, ISQ at baseline, 2, 4, and 8–12 weeks and marginal bone levels at 6 and 12 months, with survival and biological complications tracked to one year. For carbon-based and titanium nanotube systems, coupled efficacy–toxicity programs should track coating retention, particle shedding under cyclic load, and tissue distribution. For nanotube designs, at least three predefined diameters should be evaluated under otherwise identical conditions and a target dimension with a practical tolerance band declared. For ion-releasing or antimicrobial surfaces, genotoxicity should be considered, ion release quantified, and antimicrobial tests performed on a concise oral panel (e.g., Porphyromonas gingivalis, Fusobacterium nucleatum, Streptococcus mutans) under static and flow conditions, with effects normalized to surface area and exposure time. Research on ultrafine-grained and severely plastically deformed metals remain promising to validate combining mechanical resilience with hierarchical roughness, but clinical translation is still not completed developed [11,82]. Finally, meta-research should address reporting gaps by adopting a consensus for implant surface trials and by mandating complete surface characterization, enabling more decisive synthesis across nanoplatforms.

## 5. Conclusions

Nanoengineered titanium surfaces show consistent early gains in osseointegration in preclinical models, with repeatable signals for anodized titanium dioxide nanotubes, selected bioactive or ion-releasing layers, and several graphene-based coatings. These benefits are most evident in the first few weeks to months, while durable superiority over contemporary microrough controls remains uncertain in the limited and heterogeneous clinical literature. Nanofeatured titanium surfaces appear most relevant when accelerating stability is clinically decisive—namely immediate/early loading, low-density (D3–D4) bone and the posterior maxilla; short implants or augmented sites, where maximizing early bone–implant contact is advantageous; and higher-biologic risk contexts, provided the selected platform demonstrates cytocompatibility, documented antimicrobial action, and coating integrity. These indications remain hypothetical; durable superiority over contemporary microrough controls is unproven. Antibacterial functionality is a complementary strength of some nanocoatings, but translation depends on balancing antimicrobial effect with cytocompatibility and maintaining coating integrity. Standardized multiscale surface characterization, harmonized osseointegration and microbiologic endpoints, and adequately powered trials with longer follow-up are needed to define indications where nanofeatures translate into patient-important benefits.

## Figures and Tables

**Table 1:** PICO design.

Element	Definition
P—Population	Dental implants in human patients; preclinical in vivo animal models with titanium implant placement; and in vitro/laboratory models using titanium substrates relevant to dental implantology.
I—Intervention	Nanocoated or nanoscale surface-modified titanium implant/abutment surfaces (e.g., TiO_2_ nanotubes; hydroxyapatite nanoparticles; silver-based coatings; nanopatterned or micro/nanotextured surfaces; strontium, Zn/Mg ion incorporation; ultrananocrystalline diamond; nanofibers; nanospikes).
C—Comparator	Conventional or standard titanium surfaces (e.g., machined, grit-blasted/acid-etched [SLA], dual acid-etched, smooth, or uncoated titanium).
O—Outcomes	Primary osseointegration metrics (bone-to-implant contact [BIC], bone density, removal/reverse torque, bone area/volume such as BAFo, BV/TV). Secondary where applicable: ISQ, PBA-T, BMD/BMC, MAR, ALP, osteogenic gene expression, bone formation.
Study designs/models	Comparative clinical studies (including RCTs), controlled in vivo animal experiments, and in vitro/lab studies assessing osseointegration-relevant endpoints.

**Table 2:** Electronic search strategies (PubMed, Scopus, Web of Science, and Elicit/Semantic Scholar).

Database/Platform	Syntax/Search String Used	Fields Searched	Limits/Filters
PubMed (MEDLINE)	((“Dental Implants”[Mesh] OR “dental implant”[tiab] OR “oral implant”[tiab]) AND (“Nanostructures”[Mesh] OR “nanostructure *”[tiab] OR “nanoparticle *” [tiab] OR “nanotube *” [tiab] OR “nanocoating”[tiab] OR “nano coating”[tiab] OR “nanomaterial *”[tiab]) AND (“Osseointegration”[Mesh] OR osseointegration[tiab] OR “bone to implant contact”[tiab] OR BIC [tiab]))	MeSH + Title/Abstract	Years: 2005–2025; Language: English; Document type: Articles (exclude reviews, conference abstracts).
Scopus (Elsevier)	TITLE-ABS-KEY ((“dental implant” OR “oral implant”) AND (nanostructure * OR nanoparticle * OR nanotube * OR nanocoating OR “nano coating” OR nanomaterial *) AND (osseointegration OR “bone to implant contact” OR BIC))	TITLE, ABSTRACT, KEYWORDS	Years: 2005–2025; Language: English; Document type: Articles (exclude reviews, conference papers).
Web of Science Core Collection (Clarivate)	TS = ((“dental implant” OR “oral implant”) AND (nanostructure * OR nanoparticle * OR nanotube * OR nanocoating OR “nano coating” OR nanomaterial *) AND (osseointegration OR “bone to implant contact” OR BIC))	Topic (Title, Abstract, Author Keywords, Keywords Plus)	Years: 2005–2025; Language: English; Document type: Articles (exclude reviews, proceedings).
Elicit (Semantic Scholar)	Keyword query reflecting the three concepts: (i) dental/oral implants; (ii) nanoscale structures/coatings (nanoparticles, nanotubes, nanomaterials, nanocoatings); (iii) osseointegration/BIC. (Elicit converts queries to keywords and retrieves top results from the Semantic Scholar corpus via its API.)	Title/Abstract from Semantic Scholar results; full text used by Elicit when available	Years: 2005–2025; Language: English; Article-type emphasis at screening. (Elicit supports date/study-type filters.)

Notes. MeSH = Medical Subject Headings; tiab = Title/Abstract field in PubMed; TS = Topic field in Web of Science; * = truncation (captures word variants/plurals). If searches retrieved excessive orthopedic-implant literature, we applied an optional specificity filter by adding (dentistry OR oral OR periodontal) to the query.

**Table 3:** Inclusion and exclusion criteria.

Inclusion Criteria	Exclusion Criteria
Comparative studies evaluating nanocoated or nanoscale surface-modified titanium implant/abutment surfaces versus standard/conventional titanium surfaces.	Non-dental implant contexts (e.g., orthopedic-only or non-oral applications).
Original research: randomized or controlled clinical studies, controlled in vivo animal experiments, or in vitro/laboratory studies with osseointegration-relevant endpoints.	Implants or substrates made exclusively of non-titanium materials (e.g., zirconia) without a titanium-based comparator.
Reports at least one osseointegration outcome (e.g., BIC, bone density, removal/reverse torque, BV/TV/BAFo, ISQ/PBA-T, BMD/BMC, MAR, ALP, osteogenic gene expression, histomorphometry).	Reviews, editorials, letters, conference abstracts without extractable comparative data, and protocols without results.
Controlled design with an explicit comparator surface.	Surface modifications that are limited to purely microscale texturing or non-surface interventions (e.g., grafts, biologics) without a nanoscale surface modification to the titanium implant/abutment.
	Soft-tissue-only evaluations that lack bone or implant stability endpoints pertinent to osseointegration.

**Table 4:** RoB 2 (randomized clinical trials).

Study	D1 Randomization	D2 Deviations from Intended Interventions	D3 Missing Data	D4 Outcome Measurement	D5 Selection of Reported Results	Overall
Ko et al., 2024 [44]	Low	SC	SC	Low	Low	SC
Hall et al., 2019 [45]	SC	SC	SC	Low	SC	SC

Legend: Low = low risk; SC = some concerns.

**Table 5:** ROBINS-I (non-randomized human clinical studies).

Study	Confounding	Selection of Participants	Classification of Interventions	Deviations from Intended	Missing Data	Outcome Measurement	Selection of Reported Result	Overall
Karazisis et al. [46]	Mod	Mod	Low	Low	Low	Low	Mod	Mod
Zekiy et al. [47]	Ser	Mod	Low	Low	Mod	Mod	Mod	Ser

Legend: Low = low risk; Mod = moderate; Ser = serious.

**Table 6:** OHAT risk-of-bias (primary) for animal in vivo studies. Legend: ++ = definitely low; + = probably low; − = probably high; Tier = OHAT study confidence (1 highest–3 lowest).

Study (Short Title)	Randomized Exposure	Allocation Concealment	Appropriate Groups	Confounding Handled	Identical Conditions	Personnel Blinded	Outcome Data Complete	Exposure Characterization	Outcome Assessment	All Outcomes Reported	Other Issues	OHAT Tier
Scarano et al. [48]	−	−	+	−	+	−	++	+	+	−	+	2
Bierbaum et al. [49]	−	−	+	−	+	−	++	+	+	−	+	3
Wang et al. [50]	−	−	+	−	+	−	+	+	+	−	+	3
Chappuis et al. [51]	−	−	+	−	+	−	+	+	+	−	+	3
Costa-Filho et al. [52]	−	−	+	−	+	+	++	+	+	−	+	2
Hoornaert et al. [53]	−	−	+	−	+	−	+	+	+	−	+	3
Rousseau et al. [54]	−	−	+	−	+	−	+	+	+	−	+	3
Somsanith et al. [55]	−	−	+	−	+	−	++	+	+	−	+	3
Toscano et al. [56]	−	−	+	−	+	−	+	+	+	−	+	3
Li et al. [57]	−	−	+	−	+	−	+	+	+	−	+	3
Gil et al. [58]	−	−	+	−	+	−	+	+	+	−	+	3
Kang et al.—in vivo arm [59]	−	−	+	−	+	−	++	+	+	−	+	2
Lee et al.—in vivo arm [60]	−	−	+	−	+	−	+	+	+	−	+	3
Sadrkhah et al.—in vivo arm [61]	−	−	+	−	+	−	+	+	+	−	+	3
Yu et al.—in vivo arm [62]	−	−	+	−	+	−	+	+	+	−	+	3
Zhang et al.—in vivo arm [63]	−	−	+	−	+	−	++	+	+	−	+	2
Almeida et al. [64]	−	−	+	−	+	−	+	+	+	−	+	3
Das et al. [65]	−	−	+	+	+	−	+	++	+	−	+	2
Yang et al.—in vivo arm [66]	−	−	+	+	+	-	++	+	+	−	+	2

**Table 7:** RoBDEMAT (in vitro dental materials/cell and antimicrobial).

Study	Specimen/Sample Preparation	Randomization of Specimens	Blinding (Operators/Assessors)	A Priori Sample Size/Power	Outcome Validity	Replicates and Statistics	Selective Reporting	Overall
Memon et al. [67]	Low	SC	SC	High	Low	SC	SC	SC
Auciello et al. [68]	Low	SC	SC	High	Low	SC	SC	SC

Legend: Low = low risk; SC = some concerns; High = high risk.

**Table 8:** Summary of findings (GRADE)—nanocoated vs. uncoated titanium (clinical and comparative).

Outcome (Time Frame)	Studies (Design)	Participants (Approx)	Effect Signal	Certainty (GRADE)	Reason for this Rating (Downgrades/Notes)
Marginal bone loss (6–12 month)	2 RCT + 1 NR (comparative)	~75 RCT + ~60–100 NR	No consistent difference detected	Low	Risk of bias (RoB 2 “some concerns”; ROBINS-I moderate–serious) −1; imprecision (small n, underpowered; no robust pooling) −1. Direct population/comparators; short follow-up.
Soft-tissue indices (mPI/mGI/BOP; 1–6 month)	2 RCT + 1 NR	~75 RCT + ~30–60 NR	Mixed, small effects	Low	Risk of bias −1; Imprecision −1 (small samples, heterogeneous indices/timepoints). Blinded assessors in RCTs partially mitigate measurement bias.
Early infection/peri-implant mucositis	1–2 RCT ± NR	≤~75 total	Events rare; unstable	Very low	Imprecision −2 (rare events + small n); risk of bias/indirectness −1 (variable definitions).
Implant survival/“success” (≤12 month)	2 RCT + 1 NR	~75 RCT + ~30–60 NR	No clear difference; few events	Very low	Imprecision −2 (short follow-up, very low event rate); risk of bias −1; possible publication bias suspected (small studies).
Microbiological surrogates (biofilm/pathogen load)	1 RCT sub-study + NR	<~100	Direction inconsistent	Very low	Indirectness −1 (surrogate outcome), imprecision −1, risk of bias −1. Not linked to patient-important endpoints.

**Table 9:** Characteristics of included studies.

Study	Study Design	Surface Treatment Type	Control Surface	Primary Outcomes Measured
Zekiy et al., 2019 [47]	In vivo human study	Fluorine-containing nanosurface (OsseoSpeed)	Conventional titanium	Bone density
Memon et al., 2025 [67]	Combination (in vitro + in vivo human)	Silver nanoparticle (AgNP) coatings	Uncoated titanium	Bone-to-implant contact (BIC), bone density, peri-implant inflammation
Yang and Hong, 2024 [66]	Combination (in vitro + in vivo animal)	Nanostructured calcium-coated (XPEED)	Hydroxyapatite (HA), sandblasted large-grit acid-etched (SLA)	Bone-to-implant contact (BIC), bone area, removal torque
Toscano et al., 2024 [56]	In vivo animal study	Hydroxyapatite nanoparticles on zirconia-blasted/acid-etched	Zirconia-blasted/acid-etched	Reverse torque, bone area, bone-to-implant contact (BIC)
Das et al., 2019 [65]	In vivo animal study	Osteogenic nanofibrous coating	Uncoated titanium	Bone-to-implant contact (BIC), bone density, tensile strength
Chappuis et al., 2018 [51]	In vivo animal study	Hydrophilic nanopatterned (SLActive)	Commercially pure titanium (cpTi), same topography	Bone-to-implant contact (BIC), bone density, bone mineral density (BMD), bone volume/tissue volume (BV/TV)
Ko et al., 2024 [44]	In vivo human study	Hydroxyapatite nanocoated sandblasted large-grit acid-etched (SLA)	Uncoated SLA	Implant stability quotient (ISQ), periotest bone attachment time (PBA-T), bone density
Wang et al., 2022 [50]	Combination (in vitro + in vivo animal)	Titanium dioxide (TiO_2_) nanotubes, hydrogenated (superhydrophilic)	Machined titanium	Bone-to-implant contact (BIC), bone density, gene expression, alkaline phosphatase (ALP)
Scarano et al., 2020 [48]	In vivo animal study	Silver ion–TiO_2_ nanoparticle coating	Acid-etched/sandblasted	Bone-to-implant contact (BIC), bone density, bone area in threads (BAIT), bone area outside threads (BAOT)
Rousseau et al., 2021 [54]	In vivo animal study	Micro/nanofeatured (Starsurf^®^)	Standard grit-blasted	Bone-to-implant contact (BIC), bone volume
Hall et al., 2019 [45]	In vivo human study	Anodized titanium oxide (anatase)	Machined titanium	Marginal bone levels, soft tissue health
Auciello et al., 2022 [68]	In vivo animal study	Ultrananocrystalline diamond (UNCD)	Non-coated	Bone-to-implant contact (BIC), peri-implant bone area
Karazisis et al., 2021 [46]	In vivo human study	Nanopatterned (colloidal lithography)	Machined	Osteogenic gene expression
Gil and Sanz, 2025 [58]	Combination (in vitro + in vivo animal)	Nanospikes (acid/peroxide)	Grit-blasted	Bone-to-implant contact (BIC), cell adhesion, alkaline phosphatase (ALP), antimicrobial outcomes
Almeida et al., 2023 [64]	In vivo animal study	Nanostructured hydroxyapatite	Dual acid-etched	Bone-to-implant contact (BIC), bone area fraction occupied (BAFo)
Bierbaum et al., 2018 [49]	In vitro study	Nanotubular/nanopitted (anodization)	Sandblasted acid-etched (SBAE)	Alkaline phosphatase (ALP), mineralization, gene expression, bacterial adhesion
Costa-FIlho et al., 2024 [52]	Combination (in vivo animal + in vitro)	Micro–nanotextured + strontium	Machined	Bone-to-implant contact (BIC), bone volume/tissue volume (BV/TV), gene expression
Hoornaert et al., 2020 [53]	In vivo animal study	Nanostructured (grit-blasted/acid-etched)	Grit-blasted/acid-etched	Bone-to-implant contact (BIC), bone surface/tissue surface (BS/TS)
Kang et al., 2020 [59]	Combination (in vitro + in vivo animal)	Titanium dioxide (TiO_2_) nanotubes + Korean Red Ginseng extract	Nanostructured titanium (N-Ti)	Bone formation, bone mineral density (BMD), BMP-2/7, alkaline phosphatase (ALP)
Lee et al., 2019 [60]	In vivo animal study	Titanium dioxide (TiO_2_) nanotubes (anodic oxidation)	Machined	Bone coverage (qualitative)
Somsanith et al., 2018 [55]	Combination (in vitro + in vivo animal)	Propolis-loaded titanium dioxide (TiO_2_) nanotubes	Commercially pure titanium (CP-Ti)	Bone density, bone formation, alkaline phosphatase (ALP), BMP-2/7
Sadrkhah et al., 2023 [61]	In vivo animal study	SLA/SLActive on nanostructured commercially pure titanium (CP-Ti)	Unmodified CP-Ti, SLA, SLActive	Bone-to-implant contact (BIC), bone mineral density (BMD), bone mineral content (BMC), bone volume/tissue volume (BV/TV)
Li et al., 2023 [57]	Combination (in vitro + in vivo animal)	Strontium-loaded nanotextured titanium, TiO_2_ nanotubes	Each other	Bone-to-implant contact (BIC), bone density, mineral apposition rate (MAR), alkaline phosphatase (ALP)
Zhang et al., 2021 [63]	Combination (in vitro + in vivo animal)	TiO_2_ nanotubes + PLGA/rhBMP-2	Smooth titanium	Bone-to-implant contact (BIC), removal torque, gene expression
Yu et al., 2016 [62]	Combination (in vitro + in vivo animal)	Zinc/magnesium ion co-implanted titanium	Mg-PIII, Zn-PIII, pure titanium	Bone-to-implant contact (BIC), bone density, antimicrobial outcomes

**Table 12:** Antimicrobial mapping (coating ↔ organism ↔ assay).

Study	Coating Class	Strain(s) Explicitly Mentioned	Assay Type(s) Detected
Scarano et al. [48]	Ag-containing	Escherichia coli, Staphylococcus aureus, Streptococcus mutans, Streptococcus sanguinis	Bacterial adhesion (static), biofilm under flow/shear, CFU/plate counts, zone of inhibition/disk diffusion
Bierbaum et al. [49]	TiO_2_ nanotubes (TNT)	Streptococcus sanguinis	Bacterial adhesion (static), biofilm under flow/shear, CFU/plate counts, live/dead or viability staining, zone of inhibition/disk diffusion
Wang et al. [50]	TiO_2_ nanotubes (TNT)	Not specified	Bacterial adhesion (static), zone of inhibition/disk diffusion
Hall et al. [45]	TiO_2_ nanotubes (TNT)	Fusobacterium nucleatum, Porphyromonas gingivalis, Prevotella intermedia	Bacterial adhesion (static), CFU/plate counts, live/dead or viability staining, zone of inhibition/disk diffusion
Gil et al. [58]	Nanospikes/mechano-bactericidal	Actinomyces viscosus, Enterococcus faecalis, Streptococcus gordonii, Streptococcus oralis	Bacterial adhesion (static), CFU/plate counts, live/dead or viability staining, zone of inhibition/disk diffusion
Memon et al. [67]	TiO_2_ nanotubes (TNT)	Candida albicans, Pseudomonas aeruginosa, Staphylococcus aureus	Bacterial adhesion (static), zone of inhibition/disk diffusion
Somsanith et al. [55]	TiO_2_ nanotubes (TNT)	Not specified	Bacterial adhesion (static), live/dead or viability staining, zone of inhibition/disk diffusion
Toscano et al. [56]	Nano-HA/CaP-incorporated	Not specified	Zone of inhibition/disk diffusion
Li et al. [57]	TiO_2_ nanotubes (TNT)	Not specified	Live/dead or viability staining, zone of inhibition/disk diffusion
Yu et al. [62]	TiO_2_ nanotubes (TNT)	Escherichia coli, Fusobacterium nucleatum, Porphyromonas gingivalis, Staphylococcus aureus, Streptococcus mutans	CFU/plate counts, zone of inhibition/disk diffusion
Zekiy et al. [47]	F -incorporated nanosurface	Aggregatibacter actinomycetemcomitans, Porphyromonas gingivalis, Prevotella intermedia	Zone of inhibition/disk diffusion
Zhang et al. [63]	TiO_2_ nanotubes (TNT)	Streptococcus mutans	CFU/plate counts, live/dead or viability staining, zone of inhibition/disk diffusion

**Table 13:** Biological effects of nanocoated titanium—host–cell response, inflammation, antimicrobial specifics, and safety (directional, non-pooled).

Coating Class (Representative Studies)	Host-Cell/Cytocompatibility and Differentiation	Inflammatory/Immune Readouts	Antimicrobial Details (Strain·Assay·Effect)	Genotoxicity/Safety Notes	Model and Timepoints/Control
Anodized TiO_2_ nanotubes (NTs)	Osteoblast adhesion ↑; ALP, RUNX2/OCN upregulation on NTs or related nanotopographies; hydrogenated NTs show higher early BIC with osteogenic gene expression.	—	Reduced bacterial adhesion reported on certain nano-modified Ti under dynamic flow (nano-pitted/NT-adjacent designs).	No intrinsic genotox signal reported in included data; cytocompatible in vitro.	Animal 2–12 weeks; in vitro cell culture; controls typically machined/SLA.
Ca/P-incorporated (includesHA/PEO, XPEED^®^)	High cell viability; osteogenic support; early fixation advantages (BIC/BAFo) with nanostructured HA; XPEED^®^ reports improved early metrics.	—	Antimicrobial effect not consistently shown (osteogenic focus).	No genotoxicity signal reported in included set.	Animal 4–12 weeks; human early stability; controls SLA/DAE.
Ag-containing (AgNPs/Ag-doped oxides)	Cytocompatibility dose-dependent; within studied ranges, no impairment of bone healing; variable effect on osteogenic markers.	Lower IL-6 and CRP peri-implant with AgNP vs. uncoated in a clinical cohort (*p* = 0.015).	AgNP: P. aeruginosa biofilm model → bacterial adhesion ↓ (*p* = 0.009). Note: species not typical oral pathogen (surrogate for antibiofilm capacity).	Caution at high Ag dose (cytotoxicity risk in broader context); included studies used safe ranges.	Human (biopsy/short-term) + in vitro; animal rabbit model with Ag^+^ release showed no BIC gain vs. control. Controls uncoated/SLA.
Zn/Mg-modified (PEO/doped; ion co-implantation)	Pro-osteogenic ion release; osteoblast activity supported.	—	Pg/Fn/Sm (peri-implant pathogens) ↓ ~40–50% CFU vs. cp-Ti; strain- and dose-dependent.	No specific genotox signals reported; testing sparse.	In vitro + animal models; early windows (≤12 wks); control cp-Ti.
Graphene-based and bactericidal nanospikes/nanopillars	SaOs-2: adhesion ↑ (3–7 d) and ALP ↑ at 14 d on nanospikes; early BIC higher on nanospikes vs. smooth.	—	Broad panel (oral Gram±): ~70–90% bactericidal efficiency via contact-killing (membrane stress); no added biocide required.	Cytocompatibility design-dependent (ensure cell-safe spike geometry/coverage); no consistent genotox signal reported.	In vitro + in vivo (animal/human components); early weeks; control smooth/microrough Ti.
Other (Sr, F, peptide-functionalized, UNCD)	Sr-incorporated micro–nanotextures: BIC/BV/TV and osteogenic genes ↑; UNCD: biocompatible with BIC ~similar to control.	—	Not primary focus; antimicrobial performance variable or unreported.	No adverse signals reported in included summaries.	Large-animal and small-animal models; early timepoints; controls contemporary microrough.

## Data Availability

The original contributions presented in this study are included in the article. Further inquiries can be directed to the corresponding authors. This review was not registered in advance.

## Supplementary Materials

The following supporting information can be downloaded at: https://www.mdpi.com/article/10.3390/antibiotics14121191/s1, Table S1: PRISMA checklist.

## Abbreviations

The following abbreviations are used in this manuscript:

PRISMA	Preferred Reporting Items for Systematic Reviews and Meta-Analyses
PICO	Population, Intervention, Comparator, Outcomes
MeSH	Medical Subject Headings
Tiab	Title/Abstract field (PubMed search tag)
TS	Topic field in Web of Science (Title, Abstract, Author Keywords, Keywords Plus)
API	Application Programming Interface (here: Semantic Scholar API used by Elicit)
RCT/RCTs	Randomized Controlled Trial(s)
TiO_2_	Titanium Dioxide
Zn/Mg	Zinc/Magnesium (ion incorporation or co-implantation)
HA	Hydroxyapatite
SLA	Sandblasted, Large-grit, Acid-etched (implant surface)
BIC	Bone-to-Implant Contact
BAFo	Bone Area Fraction occupied
BV/TV	Bone Volume/Tissue Volume
ISQ	Implant Stability Quotient
N/A	Not Available
PBA-T	Periotest Bone Attachment Time
BMD	Bone Mineral Density
BMC	Bone Mineral Content
MAR	Mineral Apposition Rate
ALP	Alkaline Phosphatase
cpTi/CP-Ti/cp-Ti	Commercially Pure Titanium
UNCD	Ultrananocrystalline Diamond
BAIT	Bone Area In Threads
BAOT	Bone Area Outside Threads
SBAE	SandBlasted Acid-Etched (surface)
BS/TS	Bone Surface/Tissue Surface
N-Ti	Nanostructured Titanium
PLGA	Poly (lactic-co-glycolic acid)
rhBMP-2/BMP-2/7/BMPs	Recombinant human Bone Morphogenetic Protein-2; Bone Morphogenetic Proteins 2 and 7; Bone Morphogenetic Proteins
AgNP	Silver Nanoparticle(s)
Mg-PIII/Zn-PIII	Magnesium / Zinc Plasma Immersion Ion Implantation (term used in the included study; PIII not expanded in the manuscript)
RoB 2	Cochrane Risk of Bias 2 tool (for randomized trials)
ROBINS-I	Risk Of Bias In Non-randomized Studies of Interventions
RoBDEMAT	Risk of Bias tool for Dental Materials and Translational in vitro studies (as cited for in vitro)
OHAT	Office of Health Assessment and Translation risk-of-bias framework (animal/preclinical)
SYRCLE	Systematic Review Center for Laboratory animal Experimentation (RoB tool for animal studies)
ARRIVE 2.0	Animal Research: Reporting of In Vivo Experiments (reporting guideline)
CRIME-Q	The Critical Appraisal of Methodological (technical) Quality, Quality of Reporting and Risk of Bias in Animal Research
MIRIBEL	Minimum Information Reporting In Bio–Nano Experimental Literature
XPEED	Nanostructured calcium-coated implant surface (trade name)
SLActive	Hydrophilic nanopatterned/modified SLA surface (trade name)
Starsurf^®^	Commercial micro/nanofeatured implant surface (trade name)
ZiHa/Zi	Zirconia with Hydroxyapatite nanoparticles (ZiHa) versus Zirconia control (Zi), as labeled in the cited study
